# GCN2-SLC7A11 axis coordinates autophagy, cell cycle and apoptosis and regulates cell growth in retinoblastoma upon arginine deprivation

**DOI:** 10.1186/s40170-024-00361-3

**Published:** 2024-10-26

**Authors:** Dan Wang, Wai Kit Chu, Jason Cheuk Sing Yam, Chi Pui Pang, Yun Chung Leung, Alisa Sau Wun Shum, Sun-On Chan

**Affiliations:** 1 School of Biomedical Sciences, Faculty of Medicine, The Chinese University of Hong Kong, Hong Kong SAR, China; 2 Department of Ophthalmology and Visual Sciences, The Chinese University of Hong Kong, Hong Kong SAR, China; 3 Hong Kong Hub of Pediatric Excellence, The Chinese University of Hong Kong, Hong Kong SAR, China; 4 Department of Ophthalmology, Hong Kong Children’s Hospital, Hong Kong SAR, China; 5 Hong Kong Eye Hospital, Hong Kong SAR, China; 6 Department of Ophthalmology and Visual Sciences, Prince of Wales Hospital, Hong Kong SAR, China; 7 Department of Applied Biology and Chemical Technology, The Hong Kong Polytechnic University, Hong Kong SAR, China

**Keywords:** GCN2, SCL7A11, Retinoblastoma, Arginine deprivation

## Abstract

**Background:**

Arginine deprivation was previously shown to inhibit retinoblastoma cell proliferation and induce cell death in vitro. However, the mechanisms by which retinoblastoma cells respond to arginine deprivation remain to be elucidated.

**Methods:**

The human-derived retinoblastoma cell lines Y79 and WERI-Rb-1 were subjected to arginine depletion, and the effects on inhibiting cell growth and survival were evaluated. This study investigated potential mechanisms, including autophagy, cell cycle arrest and apoptosis. Moreover, the roles of the general control nonderepressible 2 (GCN2) and mechanistic target of rapamycin complex 1 (mTORC1) signaling pathways in these processes were examined.

**Results:**

We demonstrated that arginine deprivation effectively inhibited the growth of retinoblastoma cells in vitro. This treatment caused an increase in the autophagic response. Additionally, prolonged arginine deprivation induced G2 cell cycle arrest and was accompanied by an increase in early apoptotic cells. Importantly, arginine depletion also induced the activation of GCN2 and the inhibition of mTOR signaling. We also discovered that the activation of SLC7A11 was regulated by GCN2 upon arginine deprivation. Knockdown of SLC7A11 rendered retinoblastoma cells partially resistant to arginine deprivation. Furthermore, we found that knockdown of GCN2 led to a decrease in the autophagic response in WERI-Rb-1 cells and arrested more cells in S phase, which was accompanied by fewer apoptotic cells. Moreover, knockdown of GCN2 induced the constant expression of ATF4 and the phosphorylation of 70S6K and 4E-BP1 regardless of arginine deprivation.

**Conclusions:**

Collectively, our findings suggest that the GCN2‒SLC7A11 axis regulates cell growth and survival upon arginine deprivation through coordinating autophagy, cell cycle arrest, and apoptosis in retinoblastoma cells. This work paves the way for the development of a novel treatment for retinoblastoma.

**Supplementary Information:**

The online version contains supplementary material available at 10.1186/s40170-024-00361-3.

## Introduction

Retinoblastoma is an intraocular malignancy that occurs in childhood and accounts for 4% of all childhood cancers [1]. Although current treatment modalities, such as chemotherapy, are effective in some patients, they have shortcomings, including multidrug resistance, renal toxicity, and the induction of secondary cancers [2]. As a result, alternative therapeutic strategies with minimal side effects are needed. Arginine deprivation has been shown to be an effective therapeutic approach for various solid and nonsolid tumors [3]. More than 20 clinical trials have been conducted or are underway on arginine-depleting agents in cancer therapy. Many of these compounds have demonstrated excellent safety profiles in phase I/II trials [4]. In a previous study, arginine deiminase (ADI), an arginine-degrading enzyme, was shown to effectively inhibit retinoblastoma cell proliferation and induce retinoblastoma cell death in a dose-dependent manner [5]. However, the mechanisms by which retinoblastoma cells respond to arginine deprivation are not yet fully understood.

Amino acid limitation elicits signals that reprogram metabolic pathways to restore cellular amino acid homeostasis. Mammalian cells perform amino acid sensing through at least two distinct signal transduction pathways: mechanistic target of rapamycin complex 1 (mTORC1) and general control nonderepressible 2 (GCN2) [6]. mTORC1 responds to amino acids, stress, oxygen, energy, and growth factors and promotes cell growth by triggering anabolic processes or inhibiting catabolic processes and by driving cell cycle progression [7]. Under amino acid-sufficient conditions, mTORC1 localizes to the lysosome surface and phosphorylates several substrates, including eukaryotic initiation factor 4E-binding proteins (4E-BPs) and p70 S6 kinase 1 (S6K1), promoting protein translation and cell growth [8]. However, amino acid deficiency leads to inactivation of mTORC1, resulting in the shutdown of cellular catabolism and protein translation [9]. GCN2 directly senses intracellular amino acid deficiency by binding to uncharged transfer RNAs (tRNAs), which leads to the phosphorylation of eukaryotic translation initiation factor 2α (eIF2α) and increased transcription of starvation-related transcripts, including activating transcription factor 4 (ATF4) [10]. The activation of GCN2 has been reported to regulate protein translation, autophagy induction, and growth arrest [11] to cope with the loss of essential amino acids.

Arginine is one of the amino acids that can be obtained from dietary proteins, endogenous synthesis (15% of total arginine production) and body protein turnover (approximately 80% of circulating arginine) [12]. The endogenous synthesis of arginine is sufficient in healthy adults, making it a nonessential amino acid. However, in pathological situations such as cancer, growing infants, children and adults may require more arginine, making it a semiessential or conditionally essential amino acid [13]. Normal cells undergo cell cycle arrest at the G0/G1 phase and become quiescent when they are deprived of arginine [14]. In cancer cells, arginine deficiency impairs cell growth and may eventually lead to cell death. Autophagy, cell cycle arrest, and apoptosis have been shown to be associated with arginine deprivation [15]. A previous study revealed that arginine depletion in laryngeal squamous cell carcinoma for 48 h led to the accumulation of autophagosomes and light chain 3-II (LC3-II) and inactivation of the Akt/mTOR signaling pathway, resulting in arginase-induced autophagy [16]. Here, we systematically investigated the effects and signaling pathways involved in arginine deprivation in retinoblastoma.

## Materials and methods

### Cell lines and reagents

The human retinoblastoma cell lines Y79 (ATCC, HTB-18) and WERI-Rb-1 (ATCC, HTB-169) were obtained from the American Type Culture Collection (Rockville, MD, USA). Rb-YAM10 is a primary retinoblastoma cell line isolated from the right eye of a one-year-old Chinese girl [17]. The cells were maintained in RPMI 1640 medium (Gibco, 31,800–022) supplemented with 10% fetal bovine serum (FBS, Gibco, 26,140–079) and 1% penicillin–streptomycin (Gibco, 15140–122). Recombinant human arginase (rhArg) was kindly provided by Professor Thomas Leung, Hong Kong Polytechnic University.

### Antibodies

The primary antibodies used were as follows: mouse anti-PCNA (Santa Cruz, sc-56; 1/10000); rabbit anti-OTC (Abcam, ab203859; 1/1000); rabbit anti-ASS1 (Abcam, ab191165; 1/1000); mouse anti-ASL (Santa Cruz, sc-166787; 1/1000); mouse anti-GAPDH (Thermo Fisher, AM4300; 1/5000); rabbit anti-beta-actin (Abcam, ab8227; 1/2000); mouse anti-beta-actin (Abcam, ab8226; 1/5000); rabbit anti-LC3B (Cell Signaling, 2775 s; 1/1000); rabbit anti-phospho-mTOR (Cell Signaling, 2971; 1/1000); rabbit anti-mTOR (Cell Signaling, 2972 s; 1/2000); rabbit anti-phospho-ULK1 (Cell Signaling, 5869; 1/1000); rabbit anti-ULK1 (Cell Signaling, 8054; 1/1000); rabbit anti-phospho-p70S6 kinase (Cell Signaling, 9205; 1/1000); rabbit anti-p70S6 kinase (Cell Signaling, 9202 s; 1/1000); rabbit anti-phospho-4E-BP1 (Cell Signaling, 9451; 1/1000); rabbit anti-4E-BP1 (Cell Signaling, 9644; 1/1000); rabbit anti-phospho-Akt (Cell Signaling, 4060; 1/1000); rabbit anti-Akt (Cell Signaling, 4691; 1/1000); rabbit anti-phospho-GCN2 (Abcam, Ab75836; 1/1000); rabbit anti-GCN2 (Cell Signaling, 3302; 1/1000); rabbit anti-phospho-eIF2α (Cell Signaling, 3398; 1/1000); rabbit anti-eIF2α (Cell Signaling, 9722; 1/1000); rabbit anti-ATF4 (Cell Signaling, 11815; 1/1000); mouse anti-ASNS (Santa Cruz, 365809; 1/1000); rabbit anti-cyclin A (Cell Signaling, 67955 T; 1/1000); rabbit anti-cyclin B1 ( Cell Signaling, 4138 T; 1/1000); mouse anti-CDK2 (Santa Cruz, sc-6248; 1/1000); mouse anti-CDK1 (Santa Cruz, sc-6248; 1/1000); and rabbit anti-SLC7A11 (Thermo Fisher, PA1-16,893; 1/1000).

The secondary antibodies against whole IgG conjugated to horseradish peroxidase (HRP) were as follows: goat anti-rabbit (Jackson ImmunoResearch, 111–035-003; 1/10000) and goat anti-mouse (Thermo Fisher, 31431; 1/10000).

### Cell viability assay

Y79 cells, WERI-Rb-1 cells (5,000 cells/well) and Rb-YAM10 cells (1,000 cells/well) were seeded in 50 μL of complete medium in 96-well plates supplemented with various enzyme units of rhArg in another 50 μL of complete medium in each well. After seeding, the plates were incubated for 3 days to allow the cells to grow and divide in the presence of rhArg. To assess cell viability, a Cell Counting Kit-8 (CCK8, MedChemExpress, HY-K0301) was used according to the manufacturer’s instructions. Cell viability was measured by determining the absorbance at 450 nm to obtain the optical density (OD) value.

### High-performance liquid chromatography‒mass spectrometry (HPLC‒MS) analysis of the arginine content in the medium

L( +)-Arginine (Wako Chemical Co., 4987481476998) was used as a standard for HPLC–MS analysis. The analysis was performed using a C18 5 μm column (2.1 × 100 mm, Agilent Technologies, USA) and a C18 5 μm guard column (2.1 × 5 mm, Agilent Technologies, USA). The mobile phase consisted of buffer A (0.1% v/v formic acid (FA)) and buffer B (0.1% v/v FA in acetonitrile), with the ratio of A to B set at 95% to 5%. The flow rate was set to 0.30 mL/min, and the gradient conditions of the mobile phase were used. The ESI source was operated in positive ionization mode, and the MS analysis conditions were set as follows: gas temperature, 300 °C; nebulizer, 45 psi; and nozzle voltage, 500 V. All gases used were nitrogen. Quantification was performed via multiple reaction monitoring (MRM). The MRM transitions and collision energies used for arginine are listed in Table 1. To analyze the medium, 200 μL of each sample was mixed with 800 μL of HPLC-grade methanol. This mixture was vortexed and centrifuged at 10,000 × g for 5 min. The resulting supernatant was filtered through 0.22 μm syringe filters, and 10 μL of the filtrate was subjected to HPLC‒MS analysis. Data were acquired with the MassHunter Workstation Acquisition version B.06.00 and exported to Excel for further calculations.

**Table 1:** Ion transitions and instrument settings for arginine detection

Compound	Precursor (m/z)	Product (m/z)	Fragmentor (V)	CE (Collision Energy, V)
Arginine	175.1	70	90	25

### Western blotting

To prepare the cell lysate, the cells were treated with fresh 1 × RIPA buffer (Millipore, 20–188) containing phosphatase inhibitor (Roche, 04 906 845 001) and protease inhibitor (Roche, 04 693 124 001) for 30 min on ice. The quantified cellular proteins were processed and mixed well with 3 × loading buffer (BioLabs, B7703S) and heated at 95 °C for 5 min to denature the proteins and disrupt any noncovalent bonds. To analyze the protein samples, 7.5–15% sodium dodecyl sulfate‒polyacrylamide gel electrophoresis (SDS‒PAGE) gels were used, depending on the size of the protein. Electrophoresis was performed at a constant voltage of 80 V for 30 min until the band was condensed into a shape line, after which the voltage was switched to 100 V for 1.5–2 h to allow the proteins to move through the gel. The proteins were then transferred from the gel onto a polyvinylidene difluoride membrane (Advansta, L-08008–001) by tank blotting, with a constant voltage of 100 V maintained for 2 h. The membranes were blocked with 5% nonfat milk powder and probed with the desired primary antibodies overnight at 4 °C with agitation. After the membranes were washed three times in TBST (TBS with 0.1% Tween 20), they were incubated with the appropriate HRP-conjugated secondary antibody (1/5,000) for 1 h at room temperature. The proteins were visualized with enhanced chemiluminescence detection reagents (Advansta, K-12045-D50), and the expression levels were visualized with X-ray films (Advansta, L-07014–100) developed with a Kodak medical X-ray processor. To quantify the band intensity, the GBOX F3 Gel Documentation System (Syngene) was used.

In some cases, it was necessary to visualize multiple target proteins on the same membrane. To achieve this, membrane stripping was performed to remove the previous antibodies. The target membrane was incubated with stripping buffer at 50 °C for 5–10 min to remove the bound antibodies. After stripping, the membrane was blocked with 5% nonfat powdered milk and washed three times with TBST to remove any residual stripping buffer. The protein of interest was then probed again as previously described.

### Cyto-ID autophagy detection

To assess autophagy in the cells, they were treated with or without rhArg for 72 h. After treatment, the cells were stained with a Cyto-ID® Autophagy Detection Kit (Enzo Life Sciences, ENZ-KIT175-0200) according to the manufacturer’s protocol. The cells were washed twice with 1 × assay buffer and then treated with Cyto-ID® Green Dye and Hoechst 33342 at 37 °C for 30 min. After incubation, the cells were washed with 1 × assay buffer and immediately analyzed with an inverted confocal microscope (Olympus FV1200, Japan). The fluorescence intensity and autophagolysosomal puncta were analyzed with Fiji software.

### Flow cytometry analysis

Y79 and WERI-Rb-1 cells from the control and treatment groups were collected and washed twice with phosphate-buffered saline (PBS). For the cell cycle analysis, the cells were fixed overnight in ice-cold 70% ethanol at -20 °C. The fixed cells were stored at -20 °C for several weeks. To prepare for flow cytometry analysis, the fixed cells were washed twice with PBS to remove the ethanol and resuspended in 0.5 mL of PI/RNase Staining Buffer (BD Sciences, 550825) for 15 min at room temperature in the dark. After incubation, the cells were analyzed with a BD LSRII Fortessa flow analyzer (BD, USA). To assess apoptosis, two different apoptosis detection kits were used: the FITC Annexin V Apoptosis Detection Kit I (BD Biosciences, 556547) and the Annexin V-iFluor 647 Apoptosis Detection Kit (Abcam, ab219919). For the FITC Annexin V Apoptosis Detection Kit I, the cells were resuspended in 500 μL of 1 × binding buffer and then stained with annexin V (5 μL) and PI (5 μL) for 15 min at room temperature in the dark. For the Annexin V-iFluor 647 Apoptosis Detection Kit, the cells were resuspended in 200 μL of assay buffer and then stained with annexin V-iFluor 647 conjugate (2 μL) and PI (5 μL) for 40 min at room temperature in the dark. The stained cells were analyzed with a BD FACSymphony A5.2 SORP Flow Cell Analyzer (BD, USA).

### RNA-seq analysis

Y79 cells were plated in 100-mm dishes and incubated in either complete medium or medium that was depleted of arginine by rhArg for 3 days. RNA was extracted from the cells according to the protocol for the RNA extraction mini kit (Favorgen Biotech Cooperation. Taiwan). The extracted RNA was then sent to the Beijing Genomics Institute (BGI), Hong Kong, for quantitative RNA sequencing with the DNBSEQ platform (DNBSEQ Technology). The raw reads obtained from the sequencing data were filtered with SOAPnuke software developed by BGI to remove low-quality reads, adapter sequences, and contaminant sequences. The resulting clean reads were then aligned to the reference genome hg 38 from UCSC with the HISAT 2 alignment program. After mapping, the number of reads mapped to each gene was counted with the FeatureCounts tool. The counts were then used for differential expression analysis with the DESeq2 package. A log2-fold change cutoff of > 1 and padj cutoff of < 0.05 were applied to determine the differentially expressed genes (DEGs) between the arginine-depleted group and the control group.

### shRNA-mediated knockdown

Lentiviruses carrying short hairpin RNAs (shRNAs) targeting *GCN2* and *SLC7A11* were purchased from Dahong Biotechnology Co., Ltd. (Guangzhou, China). To generate lentiviral particles for transduction, 293FT cells were cotransfected with the pLKO.1-U6-scramble-EF1a-copGFP-T2A-puro vector or target shRNA vector, along with the packaging plasmids pMD2.G and psPAX2 at a ratio of 2:1:1 using Lipofectamine 3000 (Invitrogen, L3000-015) according to the manufacturer’s instructions. After transfection, the lentiviral particles were collected from the medium of the 293FT cells on Day 3. The supernatant was filtered to remove any cellular debris before being used to infect Y79 and WERI-Rb-1 cells. Positive cells were selected with 0.1 μg/mL puromycin. Images of the transduced cells were acquired with the EVOS M5000 Imaging System (Thermo Fisher, USA). shRNA constructs with the following target sequences were used (Table 2):

**Table 2:** shRNA sequences

shRNA	Target sequence (5’ to 3’)
shGCN2#1	GCGACATACTGAAGGGCAACT
shGCN2#2	GCAGAGAAGCTTCCGATAATC
shSLC7A11#1	GCAGCTAATTAAAGGTCAAAC
shSLC7A11#2	GGGCTGATTTATCTTCGATAC

### RNA extraction and quantitative real-time PCR

Total RNA was isolated according to the protocol of the RNA extraction kit (Favorgen Biotech Corp, FATRK001). The RNA was then transcribed into cDNA with SuperScript III reverse transcriptase (Applied Biosystems, 4368814). Quantitative real-time PCR was performed with SYBR Green reagents (Applied Biosystems, A25777). The CT value was determined with a ABI Quantstudio Flex Real Time PCR instrument (USA) and normalized to the β-actin gene by the ΔΔCT calculation method. The sequences of primers used are listed in Table 3:

**Table 3:** Primer information

Name	Sequence
*β-actin*	forward: 5’-GATGGCCACGGCTGCTTC-3’
	reverse: 5’-TGCCTCAGGGCAGCGGAA-3’
*COX2*	forward: 5’-CGGTGAAACTCTGGCTAGACAG-3’
	reverse: 5’-GCAAACCGTAGATGCTCAGGGA-3’
*ACSL4*	forward: 5’-GCTATCTCCTCAGACACACCGA-3’
	reverse: 5’-AGGTGCTCCAACTCTGCCAGTA-3’
*GPX4*	forward: 5’-ACAAGAACGGCTGCGTGGTGAA-3’
	reverse: 5’-GCCACACACTTGTGGAGCTAGA-3’
*FTH1*	forward: 5’-TGAAGCTGCAGAACCAACGAGG-3’
	reverse: 5’-GCACACTCCATTGCATTCAGCC-3’

### Soft agar colony formation assay

To perform a colony formation assay, WERI-Rb-1 cells were seeded in 0.3% top agar at 9,000 cells per well in 6-well plates. The cells were then cultured for 3 weeks with the addition of 250 μL culture medium twice a week. After 3 weeks, the colonies were stained overnight with 0.1% crystal violet in 10% ethanol. The colonies were then counted with Fiji software. All the assays were performed in quadruplicate.

### Statistical analysis

Statistical analysis was performed with GraphPad Prism 9.5.0. Differences among three groups or more were assessed by one-way ANOVA followed by Bonferroni’s multiple comparison test. Comparisons between two groups were assessed with the Mann‒Whitney test. *p* values are indicated as follows: ns = not significant; *, *p* < 0.05; **, *p *< 0.01; ***, *p* < 0.001; and ****, *p* < 0.0001.

For all experiments, except microscopy-based experiments, the data are presented as the means ± SEMs, and n (at least 3) corresponds to the number of experiments. For the autophagy experiments, the data are presented as medians, and n corresponds to the number of cells analyzed, with single cells considered an experimental unit in this context.

## Results

### Arginine deprivation inhibited the growth of retinoblastoma cells in vitro

**Fig. 1:**
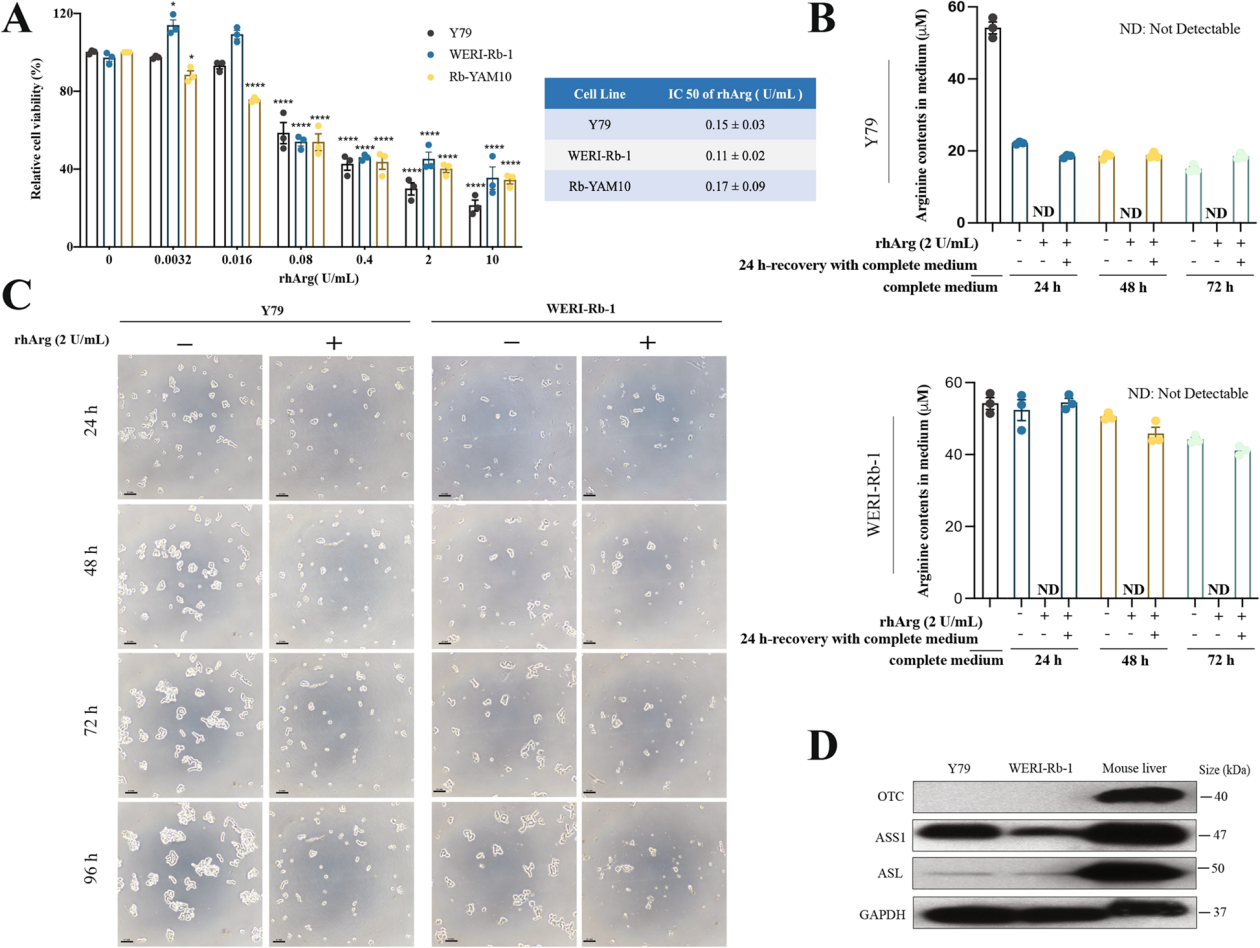
Arginine deprivation inhibited the growth of retinoblastoma cells in vitro*.* **A** Retinoblastoma cell lines (Y79, WERI-Rb-1, and Rb-YAM10) were treated with increasing concentrations of rhArg for 72 h. The data are presented as the means ± SEMs, *n* = 3. Differences were assessed by one-way ANOVA followed by Bonferroni’s multiple comparison test. *p* values are indicated as follows: *, *p* < 0.05; ****, *p* < 0.0001. **B** HPLC‒MS analysis of the arginine levels in the cell culture medium. *n* = 3. ND, not detected. **C** Growth pattern changes following arginine deprivation at the indicated time points. Scale bar, 100 μm. **D** Characterization of urea cycle enzymes in retinoblastoma. Mouse liver extract was used as the positive control

The proliferation capacity of retinoblastoma (Rb) cell lines (Y79, WERI-Rb-1, and Rb-YAM10) during arginine depletion was determined by a CCK-8 assay. After 72 h of rhArg treatment, the relative viability of Rb cells decreased significantly in a concentration-dependent manner (Fig. 1A). The 50% inhibitory concentration (IC_50_) values in Y79, WERI-Rb-1, and Rb-YAM10 cells were 0.15, 0.11 and 0.17 U/mL, respectively. To ensure the complete degradation of arginine in the medium, 2 U/mL of rhArg was used in subsequent studies. HPLC‒MS was used to quantify the arginine level in the cell culture medium. After 24 h of rhArg treatment, no arginine was detectable in any of the culture media examined (Fig. 1B). Microscopy images revealed the pattern of growth of Y79 and WERI-Rb-1 cells during the 24– to 96-h period of arginine deprivation. In the absence of arginine treatment, both cell lines grew in the form of multicellular clusters. In contrast, the cells treated with rhArg remained as single cells or small clusters, as depicted in Fig. 1C. The Rb-YAM10 cell line is a primary cell line that cannot be continuously subcultured for an extended period, which makes it unsuitable for subsequent studies. Argininosuccinate synthetase 1 (ASS1), ornithine transcarbamylase (OTC), and argininosuccinate lyase (ASL) are key enzymes that play different roles in the arginine cycle. Cancer cells lacking ASS1 and/or other arginine lyase enzymes are generally considered arginine auxotrophic tumors [18]. In our study, we analyzed the expression profiles of ASS1, OTC, and ASL in Y79 and WERI-Rb-1 cells and found that in both cell lines, OTC was undetectable, while ASS1 and ASL were expressed, but the level of ASL was minimal (Fig. 1D).

### Arginine deprivation induced autophagy, cell cycle arrest and apoptosis in retinoblastoma

**Fig. 2:**
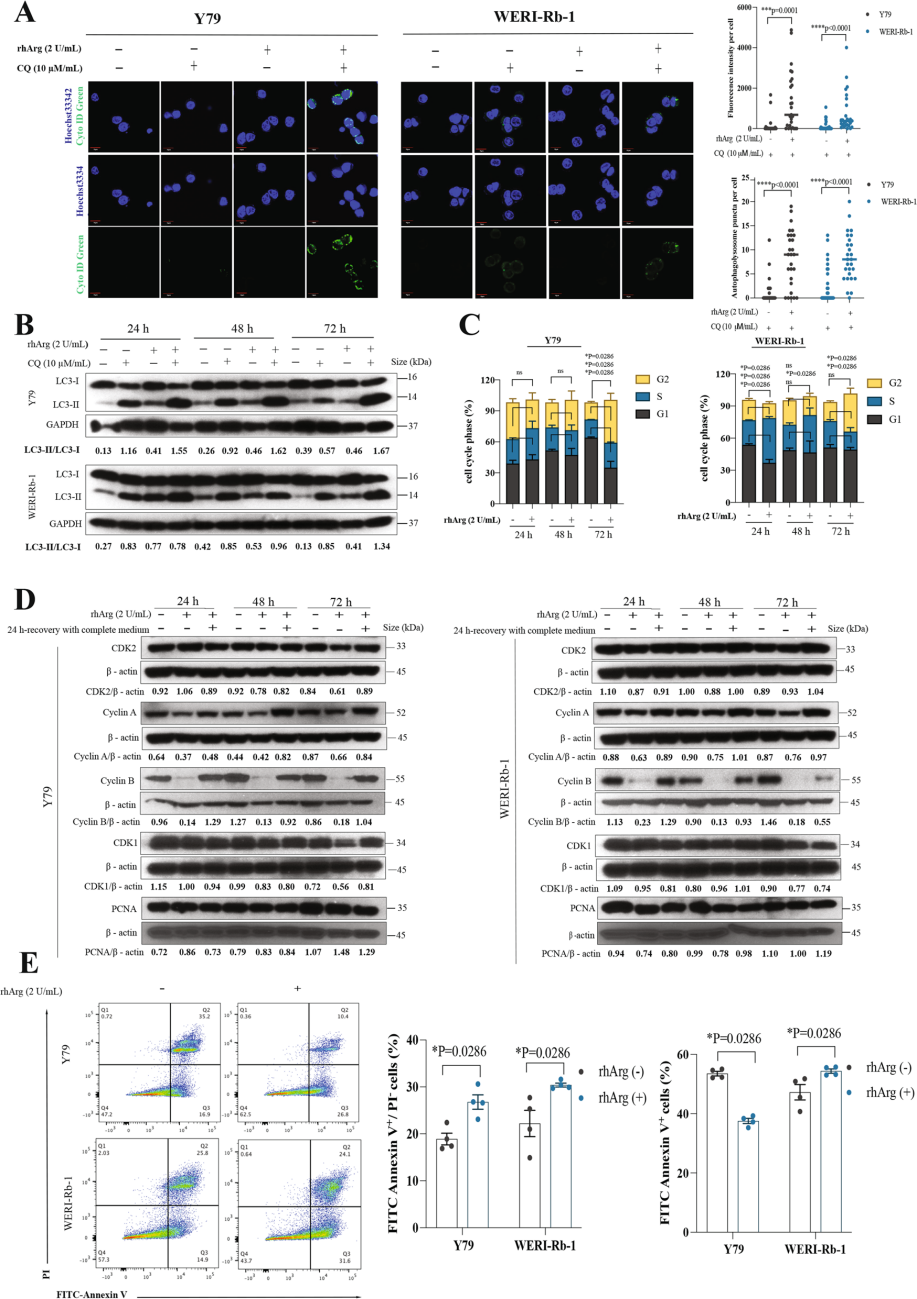
Arginine deprivation induced autophagy, cell cycle arrest and apoptosis in retinoblastoma, which were accompanied by GCN2 signaling activation and mTOR signaling inhibition. **A** Autophagy was quantified by Cyto-ID staining. Chloroquine (CQ), an autophagosome–lysosome fusion inhibitor; Hoechst 33342, stain for cell nuclei; Cyto-ID green, fluorescent dye that selectively labels accumulated autophagic vacuoles. The medians are indicated in the quantification plots. *n* = 20–27, which corresponds to the number of cells analyzed. **B** Immunoblot analyses showing time-dependent changes in autophagy activity (conversion of LC3-I to LC3-II) in Rb cells following arginine deprivation. **C** Flow cytometry analyses of cell cycle arrest. The bar chart shows the mean ± SEM of the percentage of cells in each cell cycle phase after arginine deprivation from 24– to 72 h (*n* = 4). ns = not significant.** D** Immunoblot analyses showing time-dependent changes in the expression of cell cycle-related proteins (CDK1, cyclin A, cyclin B and CDK2). **E** Flow cytometry analyses of early apoptotic cells. The bar charts show the mean ± SEM of the percentage of early apoptotic cells 72 h after arginine deprivation (*n* = 4)

Nutrient deficiency is a well-known trigger of autophagy [19]. We investigated whether arginine deprivation alone is sufficient to induce autophagy in Y79 and WERI-Rb-1 cells. The cells were treated with rhArg for 24, 48 or 72 h and then stained with Cyto-ID green dye and LC3, both of which are well-established autophagy markers. Autophagy was significantly increased in both cell lines 72 h after treatment, as evidenced by the fluorescence intensity and number of autophagolysosomal puncta per cell when the autophagosome‒lysosome fusion inhibitor chloroquine (CQ) was added under arginine-limited conditions (Fig. 2A). Moreover, increased conversion of LC3-I to LC3-II was observed in both Y79 and WERI-Rb-1 cells under arginine-deficient conditions compared with that in the control group, and this conversion was further enhanced by the addition of CQ. These findings suggested that arginine deprivation promoted significant activation of autophagy (Fig. 2B).

Cell cycle analysis revealed that, in both Y79 and WERI-Rb-1 cells, arginine restriction led to an increase in the fraction of cells in the S phase 24 and 48 h after arginine deprivation, and most cells accumulated in the G2 phase when arginine deprivation was prolonged to 72 h (Fig. 2C). Within the first 48 h, arginine deficiency had no obvious effect on the levels of CDK1 and CDK2 in either cell line. However, after 72 h, the levels of CDK1 and CDK2 decreased in Y79 cells but remained almost unchanged in WERI-Rb-1 cells (Fig. 2D). Cyclin A expression decreased slightly in both Y79 and WERI-Rb-1 cells upon arginine deprivation from 24– to 72 h (Fig. 2D). Surprisingly, cyclin B was dramatically reduced during arginine deprivation in both Y79 and WERI-Rb-1 cells, suggesting that the mitotic entry of these cells was impaired (Fig. 2D). The expression of the proliferation-related protein proliferating cell nuclear antigen (PCNA) was examined as an indirect indicator of DNA replication [20]. Surprisingly, no significant change in PCNA expression was detected in either Y79 or WERI-Rb-1 cells after arginine deprivation or in cells that recovered in complete medium (Fig. 2D). This stable expression suggested that DNA replication was not affected during arginine deprivation in retinoblastoma cells. When arginase-treated cells were replenished with complete medium for 24 h, the expression of cyclin A and cyclin B was reversed to normal levels. Overall, our cell cycle analysis revealed that cell cycle arrest in retinoblastoma cells under arginine restriction resulted directly from nutrient starvation, which caused significant impairment of mitosis.

Our next objective was to analyze whether arginine deprivation caused apoptosis and necrosis in retinoblastoma cells. We observed an increase in early apoptotic cells (annexin V^+^/PI^−^) in both Y79 and WERI-Rb-1 cells 72 h after rhArg treatment (Fig. 2E). Although the inhibition of autophagy by CQ increased the number of annexin V^+^/PI^−^ cells in both cell lines under both arginine-rich and arginine-free conditions, the difference in the number of apoptotic cells between arginine deprivation alone [rhArg(-)/CQ(-) *vs.* rhArg( +)/CQ(-)] and arginine deprivation with autophagy inhibition [rhArg(-)/CQ( +) *vs.* rhArg( +)/CQ( +)] was insignificant. These results revealed that the combination of arginine deprivation and autophagy inhibition did not lead to synergistic effects in inducing the death of retinoblastoma cells (Figure S1B).

### Arginine deprivation inhibited mTOR signaling and activated GCN2 signaling

We further examined the possible role of the mTOR signaling pathway, which is known to regulate autophagy, in arginine deprivation. However, we did not detect any significant changes in the phosphorylation of mTOR in either cell line (Figure S2A). Instead, signaling events of molecules downstream of mTOR, including the phosphorylation of p70S6K (Thr 389) and 4EBP1 (Ser 65), were strikingly inhibited, which was consistent with the observed autophagy induction. The phosphorylation of ULK1 at serine 555, which is important for autophagy initiation, was also inhibited after rhArg treatment. These results seemed to contradict our findings that arginine deprivation promoted autophagy but were consistent with other studies showing that amino acid deficiency triggers the dephosphorylation of ULK1 (Ser 555) and autophagy [21]. Interestingly, phosphorylation of Ser 473 of Akt was elevated by rhArg treatment, suggesting that the cells were struggling to survive in an unfavorable environment. To further investigate this phenomenon, we tested whether treatment with an Akt inhibitor could induce more cell death during arginine deficiency. Unfortunately, the Akt inhibitor was not effective enough to increase the number of early apoptotic control or arginase-treated cells. The differences in the number of annexin V^+^/PI^−^ cells in the rhArg(-)/Akt inhibitor VIII( +) *vs.* rhArg( +)/Akt inhibitor VIII( +) groups and in the rhArg(-)/Akt inhibitor VIII(-) *vs.* rhArg( +)/Akt inhibitor VIII(-) groups were not statistically significant (Figure S1B).

Another important nutrient deprivation-responsive pathway is the GCN2-eIF2α-ATF4 axis, which has been reported to be critical for maintaining metabolic homeostasis in tumor cells [22]. We found that under arginine deprivation in both Y79 and WERI-Rb-1 cells, the phosphorylation of GCN2 and the expression of its downstream transcription factor ATF4 were significantly activated, whereas the phosphorylation of eIF2α and the expression of the ATF4 downstream target asparagine synthetase (ASNS) were not altered (Figure S2B). Thus, although ATF4 has been widely reported as a downstream target of eIF2α, our results suggested the activation of ATF4 was not dependent on eIF2α.

### Identification of candidate genes regulated by arginine deprivation in Rb cells

**Fig. 3:**
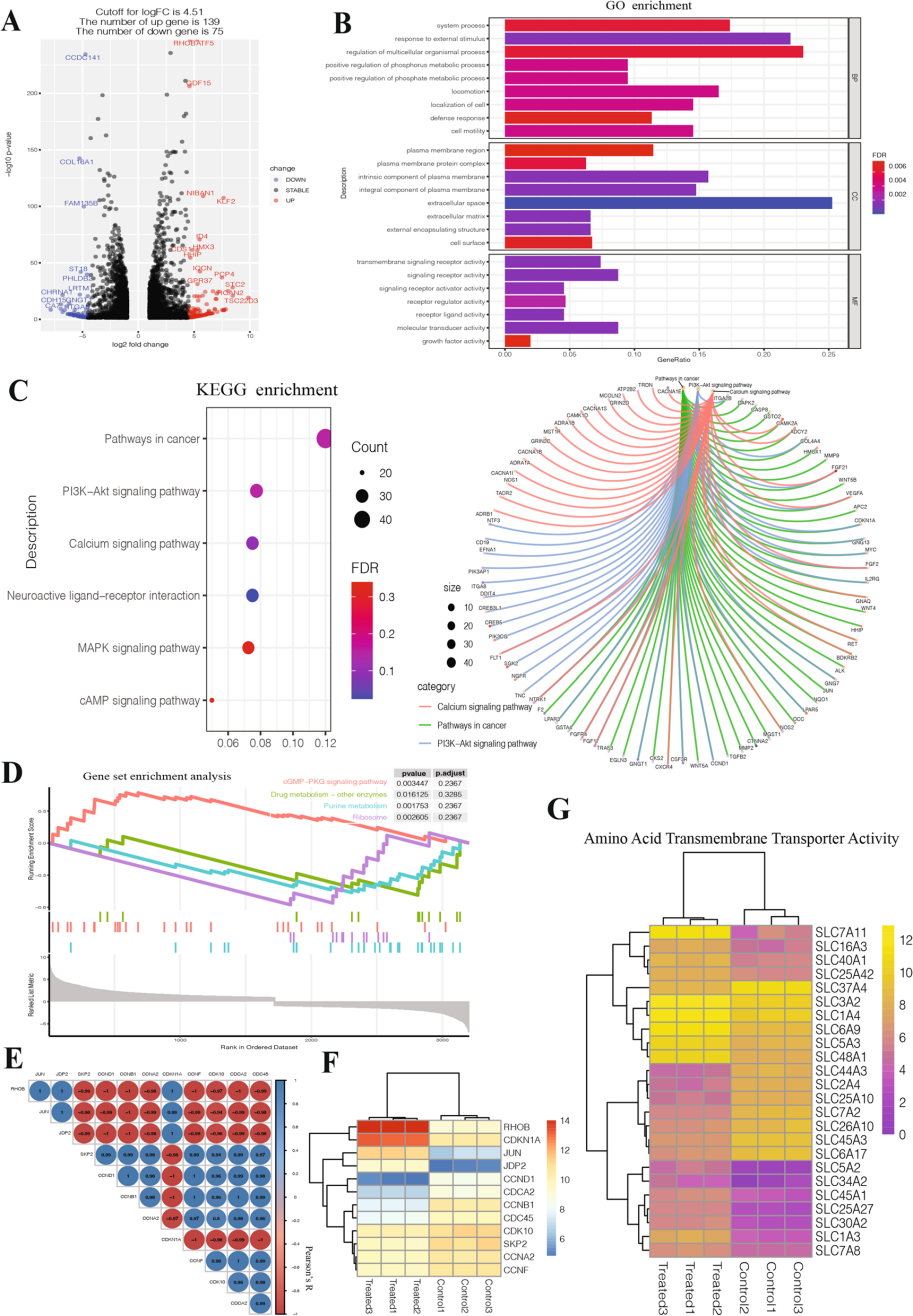
Identification of candidate genes regulated by arginine deprivation in Y79 cells. **A** Volcano plot showing changes in gene expression due to arginine deprivation. **B** and **C** GO and KEGG enrichment analyses of differentially expressed genes (DEGs) (|fold change|> 1 and adjusted *p* values < 0.05) in Y79 cells. **E** Correlations between cell cycle-related genes. **F** Heatmap of cell cycle related–related genes. **G** Heatmap of the solute carrier (SLC) family

To further elucidate the cellular response of retinoblastoma cells to arginine deprivation, RNA sequencing (RNA-seq) was used to analyze changes in gene expression in Y79 cells 72 h after rhArg treatment. Significant changes in gene expression occurred throughout the transcriptome (75 decreased and 139 increased when a log2 cutoff value with a fold change of 4.51 was applied) (Fig. 3A). Gene ontology analysis revealed that the differentially expressed genes (DEGs) were associated mainly with the response to external stimulus and regulation of multicellular organismal process under the biological process category, extracellular space under the cellular component category and signaling receptor activity and molecular transducer activity under the molecular function category (Fig. 3B). KEGG pathway analysis revealed that the DEGs were enriched in pathways related to cancer, the PI3K-Akt pathway, and the calcium signaling pathway (Fig. 3C). Gene set enrichment analysis (GSEA) revealed that the cGMP-PKG signaling pathway, purine metabolism pathway and ribosome pathway were significantly affected, although these three pathways were not significantly affected (Fig. 3D). Our results confirmed that cell cycle arrest occurred, so cell cycle-related genes were isolated from the DEGs. The correlation heatmap revealed that JUN, JDP2, RHOB, and CDKN1A were mostly negatively correlated with CCND1, CCNB1, CCNA2, CCNF, CDK10, CDCA2, and CDC45 (Fig. 3E). As most amino acids are hydrophilic and require selective transport proteins to cross the plasma membrane [23], we identified several amino acid transmembrane transporters from the solute carrier (SLC) family. A heatmap revealed that the expression levels of these transporters varied under arginine deficiency. SLC7A11 was strikingly upregulated by more than 50-fold, with a raw count log2-fold change exceeding 11 (Fig. 3G). As a result, we focused on SLC7A11 and further investigated its role in regulating Rb cell survival under arginine-deficient conditions.

### GCN2 regulated autophagy, cell cycle arrest and apoptosis in retinoblastoma upon arginine restriction

**Fig. 4:**
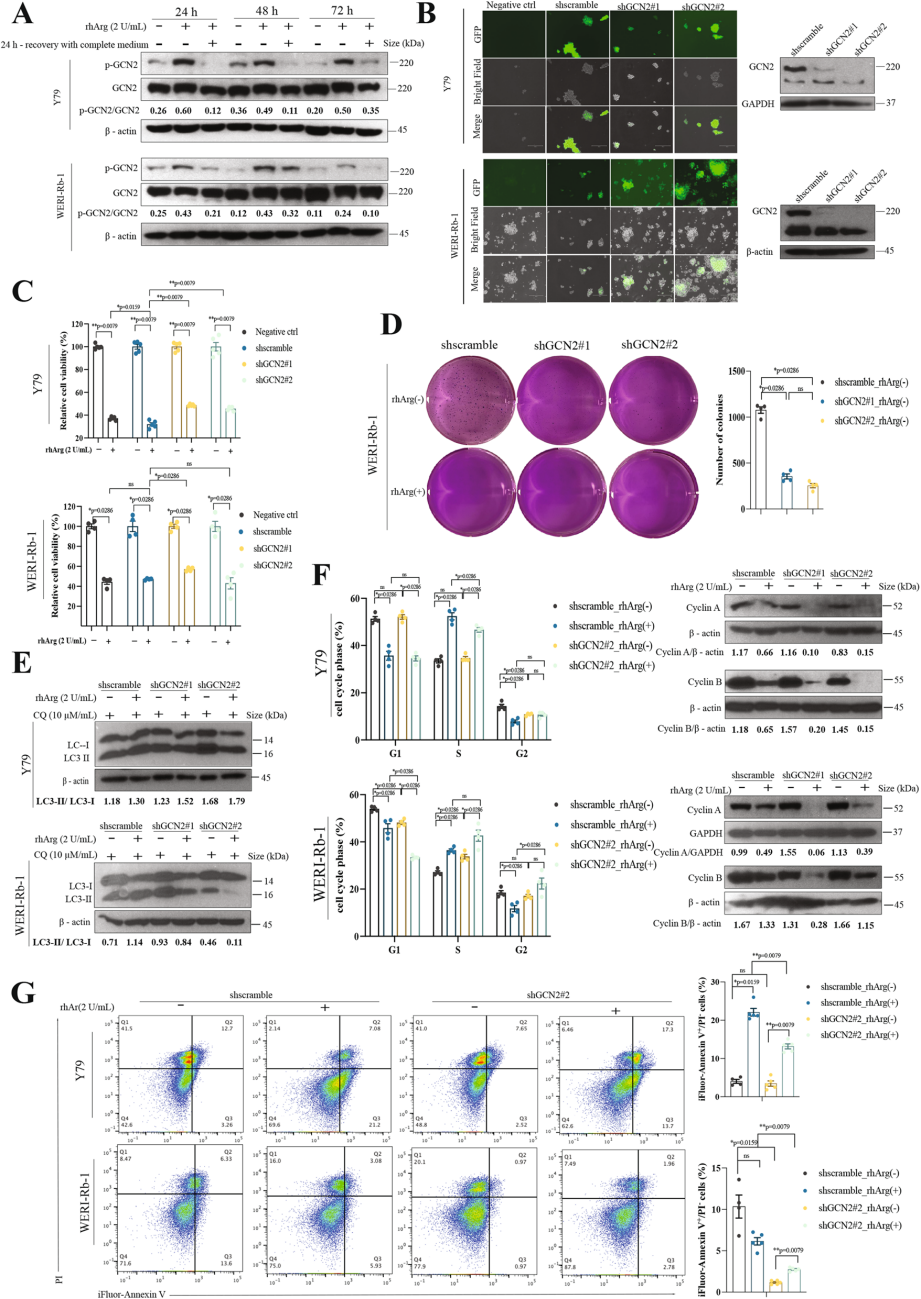
GCN2 regulated autophagy, cell cycle arrest and apoptosis in retinoblastoma upon arginine deprivation. **A** Immunoblot analyses revealed that GCN2 was induced upon arginine deprivation in both Y79 and WERI-Rb-1 cells. **B** Fluorescence microscopy image showing GFP signals in cells and immunoblot data showing the efficiency of GCN2 knockdown in Rb cells. Scale bar, 300 μm. **C** Y79 and WERI-Rb-1 cells were treated with rhArg for 72 h. The data are shown as the means ± SEMs, *n* = 4–5. Differences were assessed by the Mann‒Whitney test: ns = not significant. **D** Representative images and colony counts of GCN2-knockdown WERI-Rb-1 cells. **E** Immunoblot analyses showing the changes in autophagy activity (conversion of LC3-I to LC3-II) in Rb cells following arginine deprivation. **F** Flow cytometry analyses of cell cycle arrest. The bar chart shows the mean ± SEM of the percentage of cells in each cell cycle phase after 72 h of exposure of Rb cells to rhArg (*n* = 4). Immunoblot analyses showing the changes in cyclin A and cyclin B. **G** Flow cytometry analyses of apoptosis. The bar chart shows the mean ± SEM of the percentage of early apoptotic cells 72 h after arginine deprivation (*n* = 4)

GCN2 activity was directly induced by arginine limitation in both Y79 and WERI-Rb-1 cells (Fig. 4A). To further investigate the role of GCN2 in retinoblastoma cell growth upon arginine deprivation, we used shRNA to knock down GCN2 in Rb cells. Fluorescence microscopy revealed GFP signals within the transfected cells, indicating successful expression of the shRNA in the cells (Fig. 4B). The immunoblot data confirmed the silencing of GCN2 in both Y79 and WERI-Rb-1 cells (Fig. 4B). Analysis of cell viability in both cell lines revealed that the number of living cells was greater in the GCN2-knockdown group than in the scramble group upon arginine deprivation, suggesting that GCN2 knockdown increased cell proliferation (Fig. 4C). The soft agar colony formation assay in WERI-Rb-1 cells revealed fewer colonies in GCN2-knockdown cells than in scramble cells, confirming that the knockdown of GCN2 reduced the overall proliferation of Rb cells. Surprisingly, all the cells in the arginine-free groups died, regardless of GCN2 status, suggesting that long-term amino acid deficiency significantly impaired cell growth and that this effect was not due to the knockdown of GCN2 (Fig. 4D).

Interestingly, knockdown of GCN2 further enhanced autophagy in Y79 cells, whereas it inhibited autophagy in WERI-Rb-1 cells (Fig. 4E). According to the results of the cell cycle analysis, arginine deprivation resulted in an increased number of Y79 and WERI-Rb-1 cells arrested in S phase, whereas the knockdown of GCN2 did not significantly alter this effect. Moreover, GCN2 knockdown also induced G2 phase arrest in WERI-Rb-1 cells (Fig. 4F). The immunoblot data for cyclin A revealed a significant decrease in both Y79 and WERI-Rb-1 cells upon arginine deprivation, which was consistent with the flow cytometry data showing that the cells were arrested in S phase. The data also suggested that cyclin A was regulated by GCN2, as it did not obviously change in the cells treated with scramble shRNA upon arginine deprivation. In Y79 cells, knockdown of GCN2 significantly increased the proportion of annexin V^+^/PI^−^ cells upon arginine deprivation, similar to the results in the scramble group (Fig. 4G). However, under arginine-deprived conditions, the proportion of annexin V^+^/PI^−^ cells was significantly lower in the GCN2-knockdown group than in the scramble group, suggesting that GCN2 promoted death in Y79 cells. In WERI-Rb-1 cells, the apoptotic effects (annexin V^+^) were not significant in either the scramble group or the GCN2-knockdown group (Fig. 4G), although there were significant differences between the arginine-rich group and the arginine-free group. In both Y79 and WERI-Rb-1 cells, arginine deprivation appeared to produce an overall protective effect that rescued Rb cells from apoptotic death, which was particularly obvious in Y79 cells.

Interestingly, ATF4 was activated after GCN2 was knocked down, even in the arginine-containing group, suggesting that ATF4 is controlled not only by GCN2 but also by other unknown molecules (Figure S3A). SLC7A11 was directly induced by GCN2. The phosphorylation of 70S6K and 4E-BP1 was not decreased in GCN2-knockdown cells after arginine deprivation, suggesting that GCN2 activity may also contribute to mTOR signaling (Figure S3A). However, phosphorylated Akt (Ser 473) was still activated upon arginine limitation (Figure S3B), suggesting that Akt was not regulated by GCN2.

### SLC7A11 regulated autophagy, cell cycle arrest and apoptosis in retinoblastoma upon arginine restriction

**Fig. 5:**
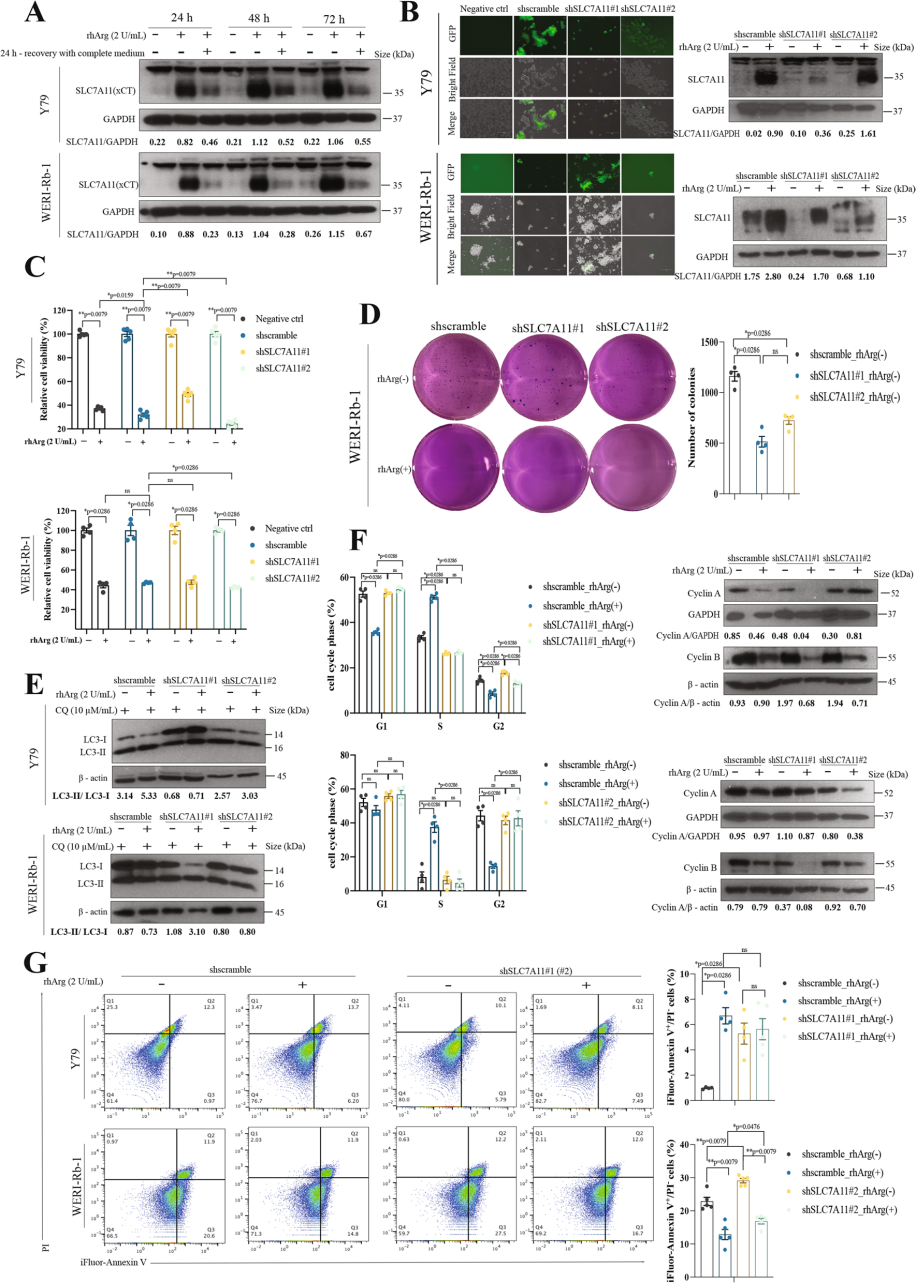
SLC7A11 regulated autophagy, cell cycle arrest and apoptosis in retinoblastoma upon arginine restriction. **A** Immunoblot analyses revealed that SLC7A11 was induced upon arginine deprivation in both Y79 and WERI-Rb-1 cells. **B** Fluorescence microscopy image showing GFP signals in the cells and immunoblot data showing the efficiency of SLC7A11 knockdown in Rb cells. Scale bar, 300 μm. **C** Retinoblastoma cell lines (Y79, WERI-Rb-1) were treated with rhArg for 72 h. The data are shown as the means ± SEMs, *n* = 4–5. Differences were assessed by the Mann‒Whitney test: ns = not significant. **D** Representative images and colony counts of SLC7A11-knockdown WERI-Rb-1 cells. **E** Immunoblot analyses showing the changes in autophagy activity (conversion of LC3-I to LC3-II) in Rb cells after arginine deprivation. **F** Flow cytometric analyses of cell cycle arrest. The bar chart shows the mean ± SEM of the percentage of cells in the cell cycle phase after 72 h of exposure of Rb cells to rhArg (*n* = 4), and immunoblot analyses revealed changes in the expression of cell cycle-related proteins (cyclin A and cyclin B). **G** Flow cytometric analyses of cell apoptosis. The bar chart shows the mean ± SEM of the percentage of early apoptotic cells 72 h after arginine deprivation (*n* = 4)

In our study, RNA-seq analysis revealed that SLC7A11 was upregulated dramatically in Y79 cells after arginine limitation, and further experiments confirmed that SLC7A11 was directly induced by arginine deprivation in both Y79 and WERI-Rb-1 cells (Fig. 5A). To investigate the role of SLC7A11 in regulating Rb cell growth upon arginine deprivation, we used shRNA to knock down SLC7A11 in Y79 and WERI-Rb-1 cells. Fluorescence microscopy revealed GFP signals in the transfected cells, and immunoblot data confirmed that shSLC7A11#1 in Y79 cells and shSLC7A11#2 in WERI-Rb-1 cells successfully knocked down SLC7A11 expression (Fig. 5B). Analysis of cell viability revealed that there was a greater percentage of living Y79 cells (shSLC7A11#1) under arginine deprivation, whereas there was a lower percentage of living WERI-Rb-1 cells (shSLC7A11#2) under the same conditions (Fig. 5C). These data suggested that SLC7A11 knockdown decreased the overall growth of Y79 cells but accelerated the growth of WERI-Rb-1 cells. A soft agar colony formation assay in WERI-Rb-1 cells revealed fewer colonies in the SLC7A11-knockdown group than in the scramble group, confirming that SLC7A11 knockdown reduced the overall growth of WERI-Rb-1 cells (Fig. 5D). Importantly, all the cells in the arginine-free groups died, indicating that long-term amino acid deficiency significantly impaired cell growth and that this effect was not caused by SLC7A11 knockdown.

Successful knockdown of SLC7A11 completely impaired autophagy regulation in Rb cells upon arginine deprivation, as the LC3-II/LC3-I ratio between the arginine-rich group and the arginine-free group was similar in both Y79 and WERI-Rb-1 cells, suggesting that SLC7A11 is necessary for the autophagic response in Rb cells upon arginine restriction (Fig. 5E).

Moreover, successful knockdown of SLC7A11 also completely impaired the cell cycle regulation of Rb cells by arginine deprivation. Both Y79 cells and WERI-Rb-1 cells no longer responded to arginine deprivation, as the cell cycle distribution remained almost the same in the SLC7A11-knockdown group compared with the scramble group (Fig. 5F). Immunoblotting data revealed that cyclin A was significantly inhibited in both Y79 and WERI-Rb-1 SLC7A11-knockdown Y79 and WERI-Rb-1 cells upon arginine deprivation. The level of cyclin B was further reduced in Y79 cells, whereas no obvious change was observed in WERI-Rb-1 cells upon arginine deprivation (Fig. 5F). In Y79 cells, arginine deprivation significantly increased the percentage of annexin V^+^/PI^−^ cells in the scramble group, but SLC7A11-knockdown cells failed to induce annexin V^+^/PI^−^ cells even in an arginine-limited environment (Fig. 5G). In WERI-Rb-1 cells, the apoptotic effects (annexin V^+^) were not significant (Fig. 5G), and the number of annexin V^+^/PI^−^ cells decreased in both the scramble group and the SLC7A11-knockdown group.

Similar to that observed in the scramble group, SLC7A11 knockdown significantly affected GCN2 and mTOR signaling. In GCN2 signaling, both GCN2 and ATF4 were activated upon arginine deprivation in both Y79 and WERI-Rb-1 cells (Figure S4A). The level of phosphorylation of 70S6K and 4E-BP1 was decreased (Figure S4B). However, phosphorylated Akt (Ser 473) was still obvious (Figure S4B) upon arginine limitation, indicating that Akt was not regulated by SLC7A11.

The role of SLC7A11 as a key component in mediating ferroptosis was demonstrated by the mRNA levels of cyclooxygenase-2 (COX-2), acyl-CoA synthetase long-chain family member 4 (ACSL4), glutathione peroxidase 4 (GPX4), and ferritin heavy chain 1 (FTH1). Although COX-2 and ACLS4 levels were elevated upon arginine deprivation, the level was significantly lower in the SLC7A11-knockdown group than in the scramble group in both Y79 and WERI-Rb-1 cells, indicating attenuation of ferroptosis (Figure S4C). GPX4 was decreased in Y79 cells but increased in SLC7A11-knockdown cells after arginine limitation. In contrast, GPX4 was increased in WERI-Rb-1 cells but decreased in SLC7A11-knockdown cells after arginine limitation, suggesting the differential regulation of lipid oxidation damage in Y79 and WERI-Rb-1 cells (Figure S4C). In the scramble group, FTH1 did not present any changes in Y79 cells response to arginine deprivation, but it was significantly increased in Y79 cells after SLC7A11 was knocked down, whereas arginine deprivation led to significant activation of FTH1 in WERI-Rb-1 cells, and knockdown of SLC7A11 prevented the regulation of FTH1 in WERI-Rb-1 cells (Figure S4C).

## Discussion

Multiple studies have examined the anticancer properties of amino acid restriction, particularly arginine deprivation, in various cancers. Despite this, only one study has specifically focused on retinoblastoma [5], making our research among the first few comprehensive investigations into the effects of arginine deprivation on this particular cancer. While previous studies on other tumors have suggested potential mechanisms and effects, our findings provide novel insights into the impact of arginine deprivation on retinoblastoma.

Our analysis of cell viability led us to conclude that nutrient deficiency induces a quiescent state in retinoblastoma cells. Previous studies have shown that some other cell lines can form colonies under arginine deprivation, such as in MCF-7 cells with a 50% knockdown of ASS1 [24] and BxPC-3 and MIA PaCa-2 cells grown in glutamine-free media [25]. However, in our colony formation assay, we observed that no colony formation occurred despite the knockdown of certain genes following arginine deprivation. These findings suggest that arginine depletion may represent a promising avenue for the treatment of retinoblastoma.

Our study revealed that autophagy, cell cycle arrest, and apoptosis were induced in retinoblastoma cells subjected to arginine deprivation, and we observed correlations among these three processes. Specifically, prolonged arginine deprivation led to G2 cell cycle arrest and an increase in early apoptotic cells. However, notably, the Y79 and WERI-Rb-1 cell lines presented different responses to arginine depletion. This discrepancy may be due to the different characteristics of the two cell lines. Y79 cells resemble invasive and metastatic human retinoblastoma, whereas WERI-Rb-1 cells more closely resemble nonmetastatic human retinoblastoma [26]. These responses could be beneficial or detrimental to cell growth. Autophagy was significantly enhanced upon arginine deprivation in both Y79 and WERI-Rb-1 cells. However, autophagy seemed to play a different role in Y79 and WERI-Rb-1 cells, as annexin V^+^ cells were decreased in Y79 cells but increased in WERI-Rb-1 cells after the inhibition of autophagy with CQ compared with those in the control group, suggesting that autophagy promotes death in Y79 cells and survival in WERI-Rb-1 cells 72 h after arginine deprivation. The prosurvival role of autophagy is relatively common, as the inhibition of autophagy has been found to synergize with low leucine concentrations in the induction of apoptosis in melanoma cells [27]. With respect to cell cycle arrest, cyclin B was dramatically inhibited during arginine deprivation in both Y79 and WERI-Rb-1 cells, indicating that the mitotic ability of these cells was significantly impaired upon arginine deprivation. The cell cycle distribution of Y79 and WERI-Rb-1 is similar to that of other tumors, such as A375 human melanoma, upon arginine deprivation [28]. Cell cycle arrest at the G2 phase normally indicates that damage to intracellular DNA is difficult to repair [29]. The inhibition of mTORC1 signaling by amino acid deprivation has been the subject of intense studies, for which there is ample evidence [30, 31]. In our study, both 70S6K and 4EBP1 were inhibited, indicating the inhibition of protein synthesis [32]. As a prosurvival kinase, the phosphorylation of Akt at Ser 473 was activated, suggesting that the cells attempted to survive in this unfavorable environment [33]. GCN2 and its downstream transcription factor ATF4 were significantly activated, whereas eIF2α showed no apparent change, suggesting that the activation of ATF4 was independent of eIF2α under our experimental conditions. When we replenished the arginine-deprived cells with complete medium for only 24 h, all GCN2 and mTOR signaling was reversed, indicating that GCN2 activation and/or mTOR inhibition were directly due to arginine deprivation; similar results also reported that proline supplementation was able to reverse GCN2 activation [34].

Successful knockdown of GCN2 increased autophagy in Y79 cells but significantly inhibited autophagy in WERI-Rb-1 cells. These findings suggest that GCN2 mediated autophagic effects of WERI-Rb-1 cells. Similar to the findings in WERI-Rb-1 cells, one study showed that the LC3B ratio and p62 level were significantly lower in GCN2^−/−^ cells than in wild-type mice, indicating a reduced autophagy flux [35]. Our study revealed that the knockdown of GCN2 did not significantly affect the cell cycle distribution of either Y79 or WERI-Rb-1 cells. However, we observed that lentivirus transfection resulted in S phase arrest rather than G2 phase arrest during the same period of arginine depletion compared with non-arginine-depleted Rb cells. This finding was consistent with the downregulation of cyclin A. Interestingly, even when GCN2 was knocked down, ATF4 was still activated. In other words, arginine deprivation led to the activation of other kinases, such as PERK, to compensate for GCN2 deficiency [36]. The trend of SLC7A11 expression was in agreement with that of GCN2, suggesting that SLC7A11 expression is controlled by GCN2. However, these findings contradicted the results of other studies that showed that the induced SLC7A11 expression was largely mediated by ATF4 [37]. Although one study showed that glucose-restricted conditions can also induce the overexpression of SLC7A11 [38], it is still unknown how GCN2 can bypass eIF2α and ATF4 to directly regulate SLC7A11 expression. The phosphorylation of 70S6K and 4E-BP1 was not decreased in GCN2-knockdown cells after arginine deprivation, suggesting that GCN2 activity also contributes to the mTOR signaling pathway. Similarly, no effect on 70S6K and 4E-BP1 phosphorylation was observed in GCN2 knockout or PERK knockout mice [39]. In summary, GCN2, as well as autophagy, promotes death at Y79 cells but promotes survival in WERI-Rb-1 cells.

In our experiments, we observed that successful knockdown of SLC7A11 did not result in any response to arginine deprivation in terms of autophagy regulation or cell cycle distribution. Moreover, we found that SLC7A11 had no significant effect on GCN2 signaling or mTOR signaling, similar to the findings in the scramble shRNA-treated group, which suggests that SLC7A11 is a downstream factor of GCN2 or mTOR and does not act as a feedback signal. Given that SLC7A11 is well known for its regulation of ferroptosis, we measured the expression of ferroptosis-related genes to determine whether ferroptosis is involved under conditions of arginine deprivation. We observed that in SLC7A11-knockdown Y79 and WERI-Rb-1 cells, the upregulation of both COX-2 and ACSL4 was attenuated. These findings suggest that the induction of ferroptosis occurs upon arginine depletion. However, the expression of ACSL4 is complicated, as ACSL4 is not only elevated during ferroptosis but also required for cells to undergo ferroptotic cell death in leukemia and liver cancer cells [40, 41]. GPX4 repairs oxidative damage to lipids and is a leading inhibitor of ferroptosis [42]. We observed different changes in GPX4 levels in Y79 and WERI-Rb-1 cells upon arginine deprivation, which suggests differential regulation of oxidative lipid damage in these cells. Another marker, FTH1, is responsible for maintaining the balance of iron metabolism in cells and consequently influences susceptibility to ferroptosis both in vitro and in vivo [43]. Activated FTH1 in Y79 cells and stable expression of FTH1 in WERI-Rb-1 cells upon arginine deprivation indicate the inhibition of ferroptosis in Y79 cells and the differential iron requirements in Y79 and WERI-Rb-1 cells. Taken together, the levels of COX-2, ACSL4, GPX4, and FTH1 in Y79 and WERI-Rb-1 cells upon arginine deprivation indicate that SLC7A11 mediates ferroptosis and that SLC7A11 knockdown attenuates ferroptosis. However, this conclusion needs to be further confirmed, as SLC7A11-mediated cysteine uptake may act as a double-edged sword in the context of cellular redox regulation [44]. Studies have shown that SLC7A11 can promote cell death in glucose-starved cells [45].

## Conclusion

**Fig. 6:**
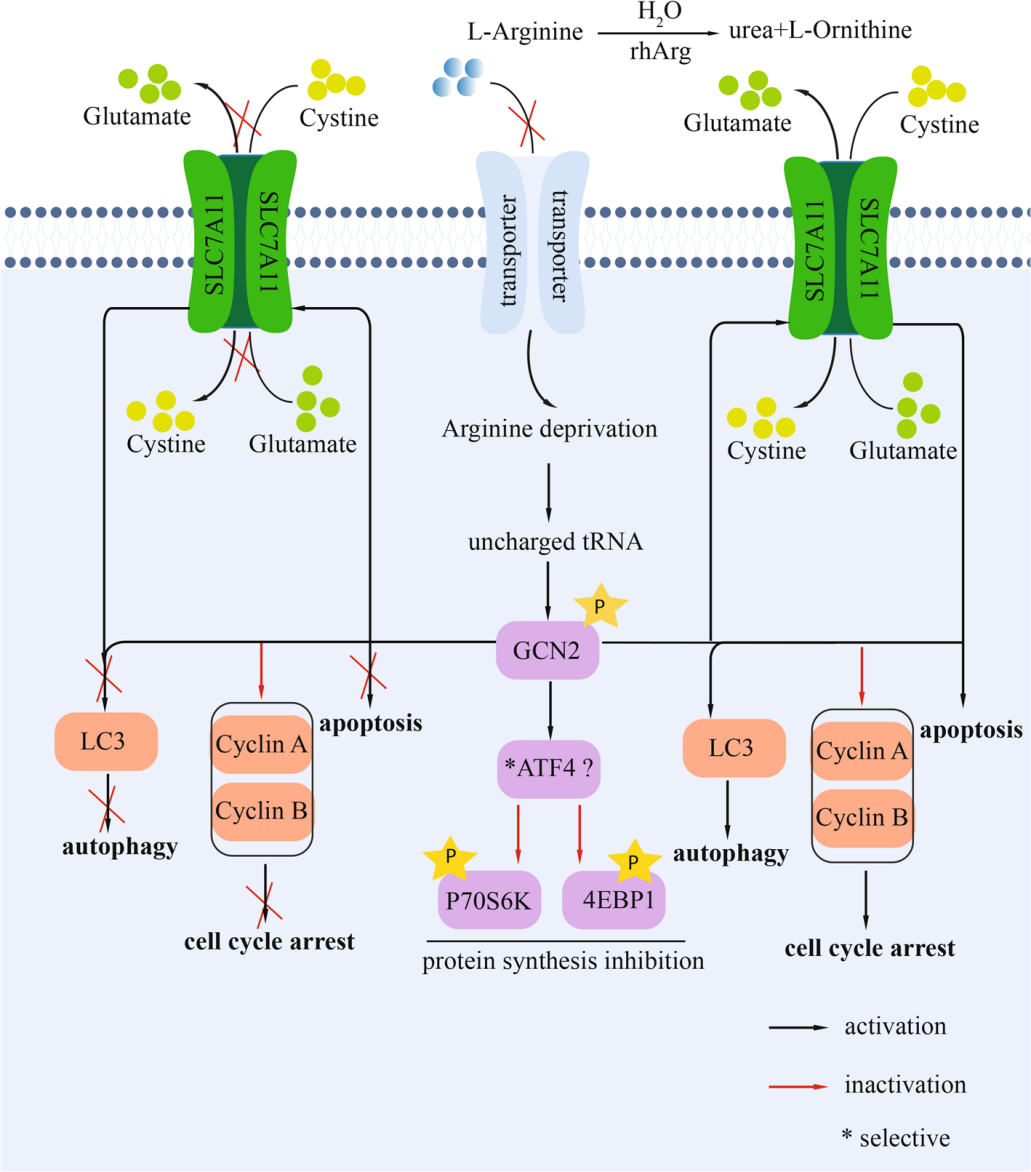
Schematic summary. Mechanisms underlying the role of GCN2-SLC7A11 in regulating cell growth and survival through autophagy, cell cycle arrest and apoptosis in retinoblastoma cells upon arginine deprivation. rhArg metabolizes arginine to H_2_O and ornithine, and arginine cannot be transported to the cell by transporters, resulting in arginine deprivation in cells. The accumulation of uncharged cognate tRNAs by arginine deprivation activates GCN2, leading to the activation of RhoB and SLC7A11 or the inhibition of 70S6K and 4E-BP1, suggesting the inhibition of protein synthesis

In conclusion, we investigated the role of GCN2 and SLC7A11 in regulating autophagy, cell cycle arrest, and apoptosis and their influences on the GCN2 and mTOR signaling pathways. Our results suggest that arginine deprivation caused an increase in the autophagic response. Moreover, prolonged arginine deprivation induced G2 cell cycle arrest, which was accompanied by an increase in early apoptotic cells. Importantly, arginine depletion also caused the activation of GCN2 and the inhibition of mTOR signaling. We also discovered that the activation of SLC7A11 was fully regulated by GCN2 upon arginine deprivation, but individual knockdown of SLC7A11 rendered retinoblastoma cells resistant to arginine deprivation. Furthermore, we found that knockdown of GCN2 led to a decrease in the autophagic response in WERI-Rb-1 cells and arrested more cells in S phase, which was accompanied by fewer apoptotic cells. Moreover, knockdown of GCN2 induced the constant expression of ATF4 and the phosphorylation of 70S6K and 4E-BP1 regardless of arginine deprivation. However, the knockdown of all these genes did not affect the activation of Akt, suggesting that the cells attempted to survive in an unfavorable environment. This work paves the way for the development of novel treatments for retinoblastoma and other tumors. The mechanisms underlying the role of GCN2-SLC7A11 in regulating cell growth and survival through autophagy, cell cycle arrest and apoptosis in retinoblastoma cells upon arginine deprivation are summarized in Fig. 6.

## Supplementary Information

Supplementary Material 1: Supplementary Figure S1. Analysis of cell cycle arrest and apoptosis in retinoblastoma upon arginine deprivation. A. Representative flow plots of propidium iodide (PI) assessment of the cell cycle phase distribution in Rb cell lines cultured under control and arginine-free conditions. B. Representative flow plots of PI and FITC-annexin V assessment of cell apoptosis distribution in Rb cell lines cultured under control and arginine-free conditions.

Supplementary Material 2: Supplementary Figure S2. mTOR signaling was inhibited, whereas the GCN2 pathway was activated in retinoblastoma upon arginine deprivation. A. Immunoblot analyses showing the inhibition of mTOR signaling in Rb cells upon arginine deprivation. B. Immunoblot analyses showing activation of the GCN2 pathway in Rb cells upon arginine deprivation.

Supplementary Material 3: Supplementary Figure S3. Regulation of GCN2 and mTOR signaling in GCN2-knockdown cells upon arginine deprivation. A. Immunoblot analyses showing the activation of GCN2 signaling in GCN2-knockdown Rb cells upon arginine deprivation. B. Immunoblot analyses showing the inhibition of mTOR signaling in GCN2-knockdown Rb cells upon arginine deprivation.

Supplementary Material 4: Supplementary Figure S4. Regulation of GCN2 and mTOR signaling in SLC7A11-knockdown cells upon arginine deprivation. A. Immunoblot analyses showing the activation of GCN2 signaling in SLC7A11-knockdown Rb cells upon arginine deprivation. B. Immunoblot analyses showing the inhibition of mTOR signaling in SLC7A11-knockdown Rb cells upon arginine deprivation. C. q-PCR analysis: COX-2, ACSL4, GPX4, and FTH1 expression levels in SLC7A11-knockdown Y79 and WERI-Rb-1 cells upon arginine deprivation.

Supplementary Material 5.

## Data Availability

No datasets were generated or analysed during the current study.

## Abbreviations

ACSL4: Acyl-CoA synthetase long chain family member 4
ADI: Arginine deiminase
ASS1: Argininosuccinate synthase 1
ASL: Argininosuccinate lyase
ASNS: Asparagine synthetase
ATF4: Activating transcription factor 4
CDKs: Cyclin-dependent kinases
COX-2: Cyclooxygenase-2
CQ: Chloroquine
DEGs: Differentially expressed genes
eIF2α: Eukaryotic translation initiation factor 2α
4E-BPs: Eukaryotic initiation factor 4E-binding proteins
FTH1: Ferritin heavy chain 1
GCN2: General control nonderepressible 2
GPX4: Glutathione peroxidase 4
GSEA: Gene set enrichment analysis
HPLC–MS: High-performance liquid chromatography‒mass spectrometry
LC3: Light chain 3
mTOR: Mechanistic target of the rapamycin complex
MRM: Multiple reaction monitoring
OD: Optical density
OTC: Ornithine carbamoyltransferase
PCNA: Proliferating cell nuclear antigen
q-PCR: Quantitative real-time polymerase chain reaction
Rb: Retinoblastoma
RT: Reverse transcription reaction
rhArg: Recombinant human arginase 1
S6K1: S6 kinase 1
SLC: Solute carrier
shRNAs: Short hairpin RNAs
tRNAs: Transfer RNAs
ULK1: Unc-51-like autophagy-activating kinase 1
