# Supplementary figures and images for: GCN2-SLC7A11 axis coordinates autophagy, cell cycle and apoptosis and regulates cell growth in retinoblastoma upon arginine deprivation
Source: Cancer Metab. 2024 Oct 26;12:31. doi: 10.1186/s40170-024-00361-3 (PMC11515237; doi:10.1186/s40170-024-00361-3)

A

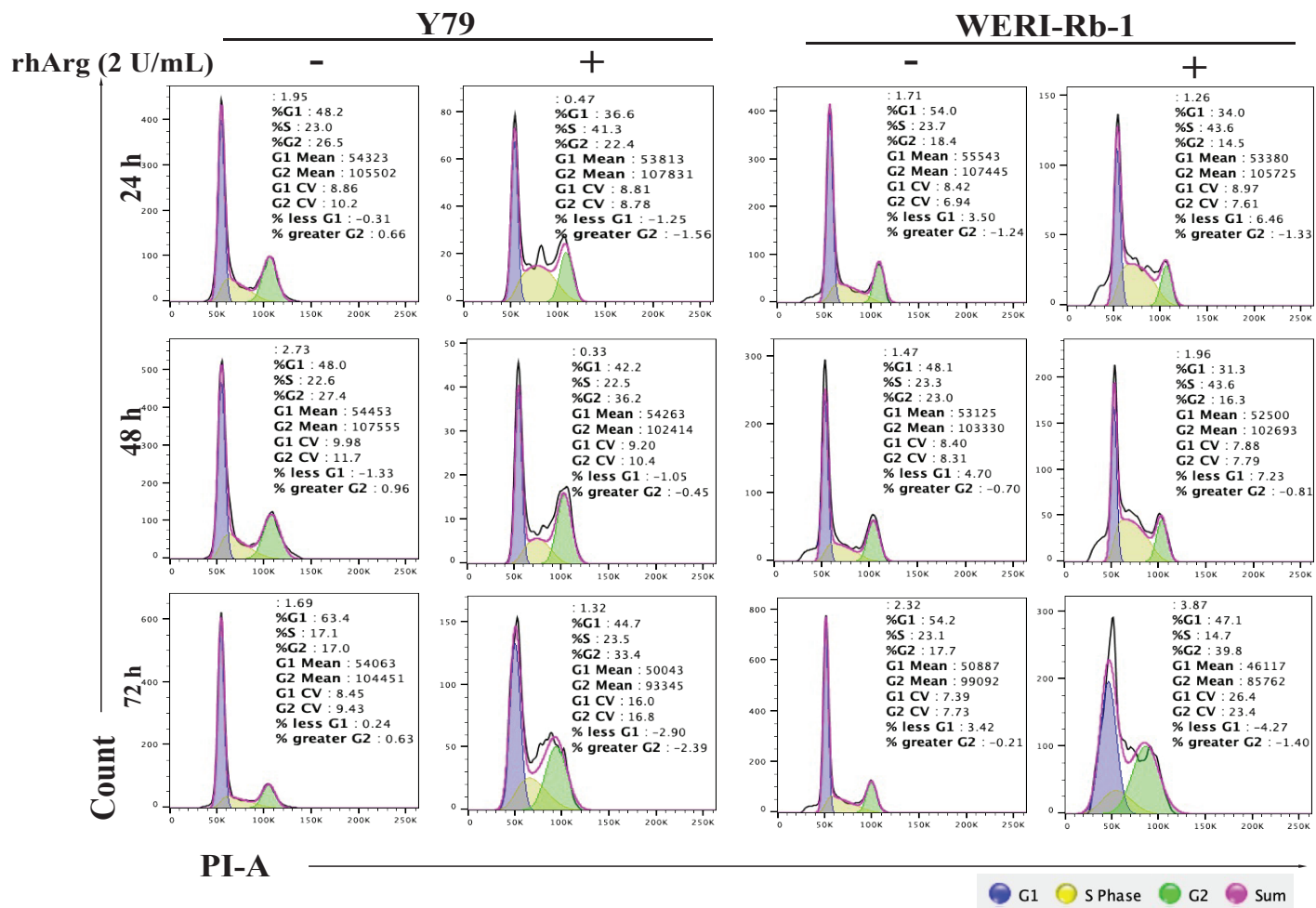

B

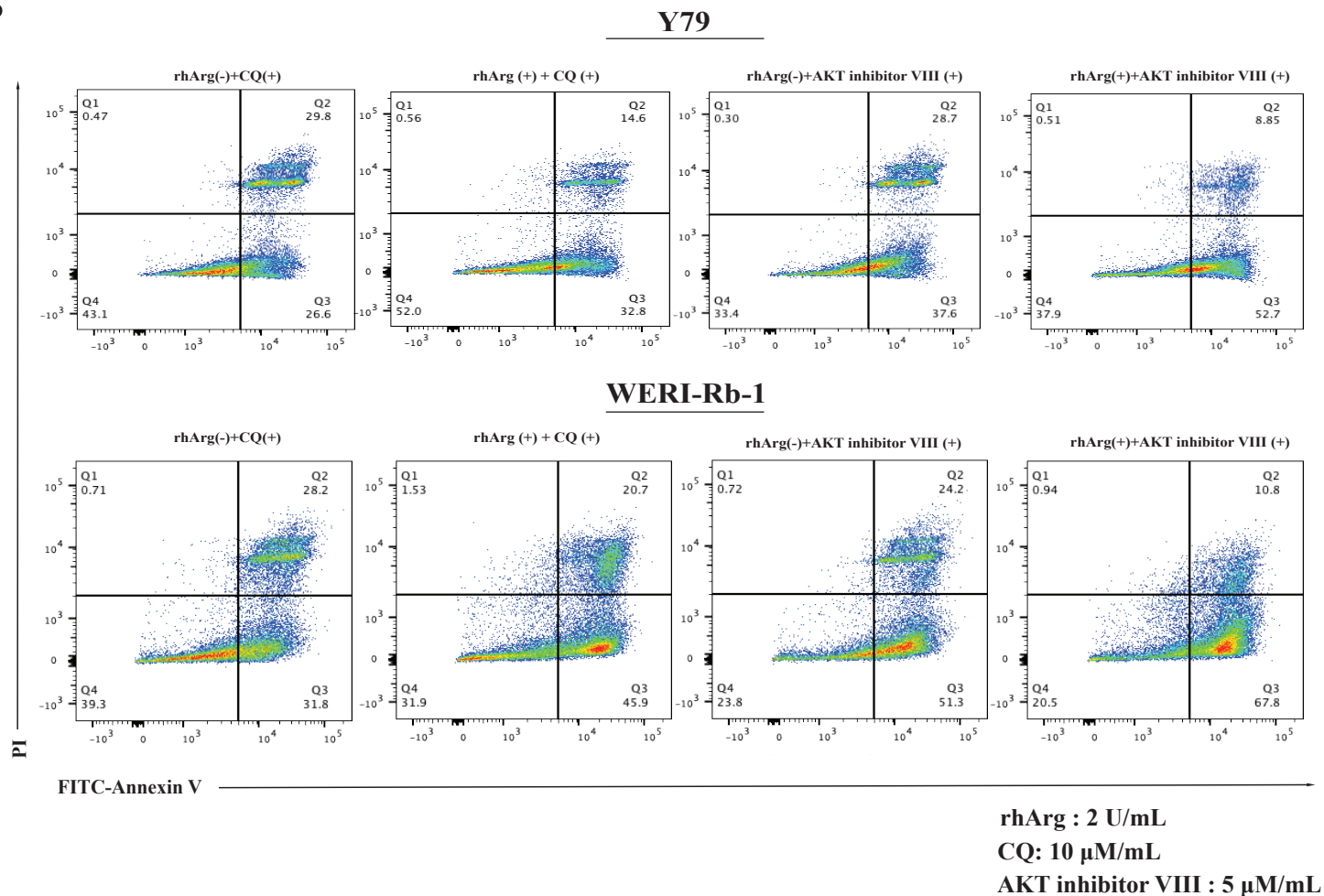

Supplement: Supplementary file 1 — Supplementary Material 1: Supplementary Figure S1. Analysis of cell cycle arrest and apoptosis in retinoblastoma upon arginine deprivation. A. Representative flow plots of propidium iodide (PI) assessment of the cell cycle phase distribution in Rb cell lines cultured under control and arginine-free conditions. B. Representative flow plots of PI and FITC-annexin V assessment of cell apoptosis distribution in Rb cell lines cultured under control and arginine-free conditions. [file 40170_2024_361_MOESM1_ESM.pdf]

A

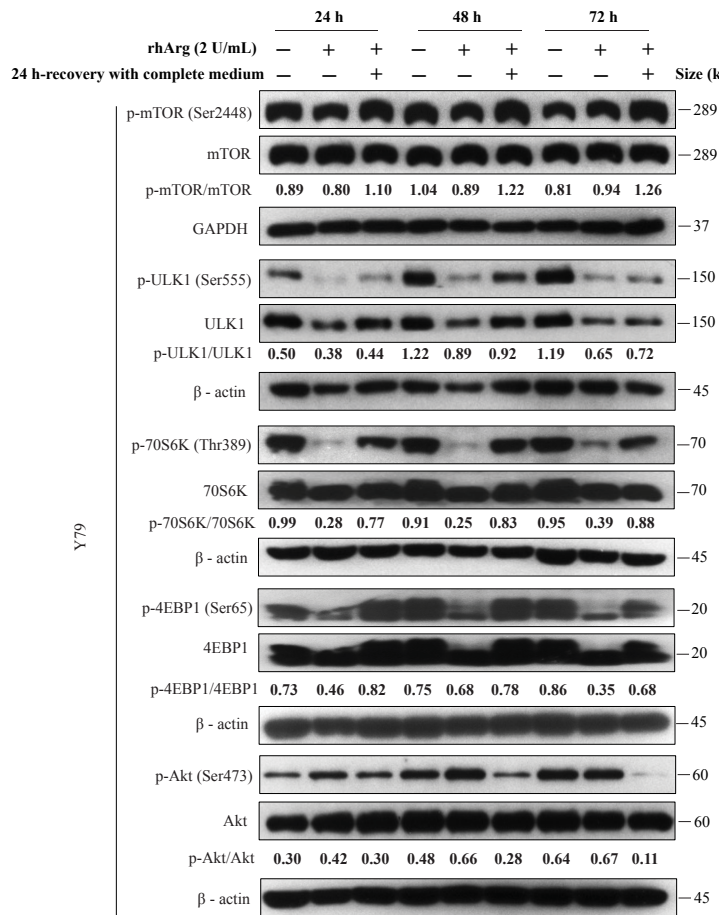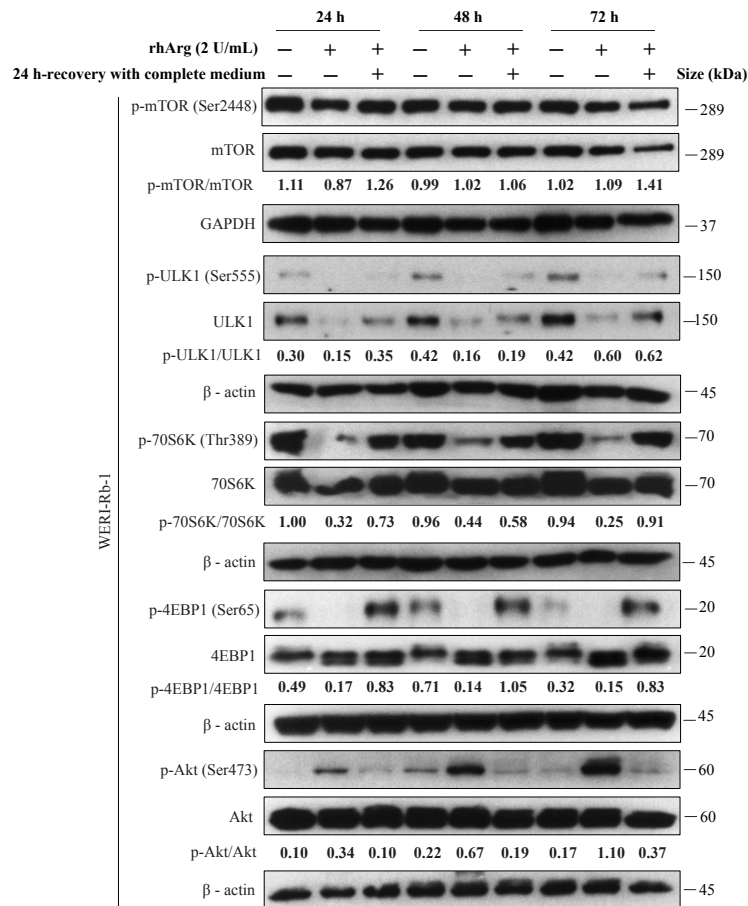

B

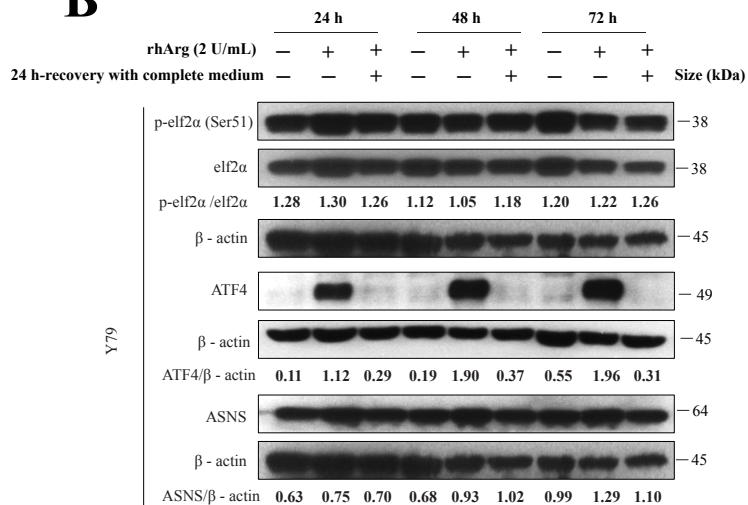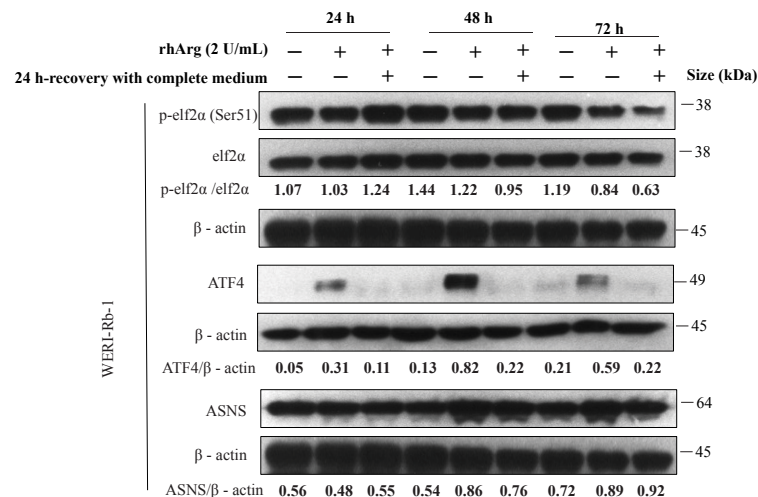

Supplement: Supplementary file 2 — Supplementary Material 2: Supplementary Figure S2. mTOR signaling was inhibited, whereas the GCN2 pathway was activated in retinoblastoma upon arginine deprivation. A. Immunoblot analyses showing the inhibition of mTOR signaling in Rb cells upon arginine deprivation. B. Immunoblot analyses showing activation of the GCN2 pathway in Rb cells upon arginine deprivation. [file 40170_2024_361_MOESM2_ESM.pdf]

**A**

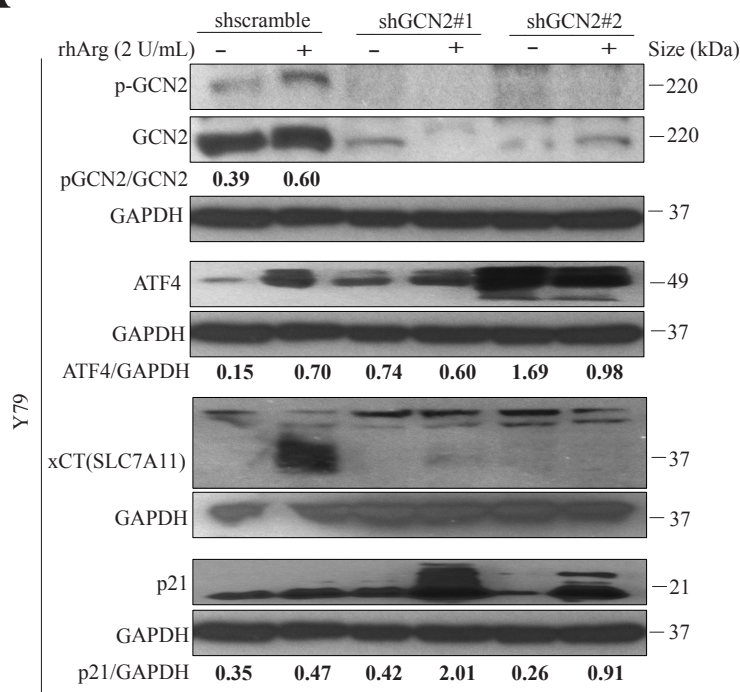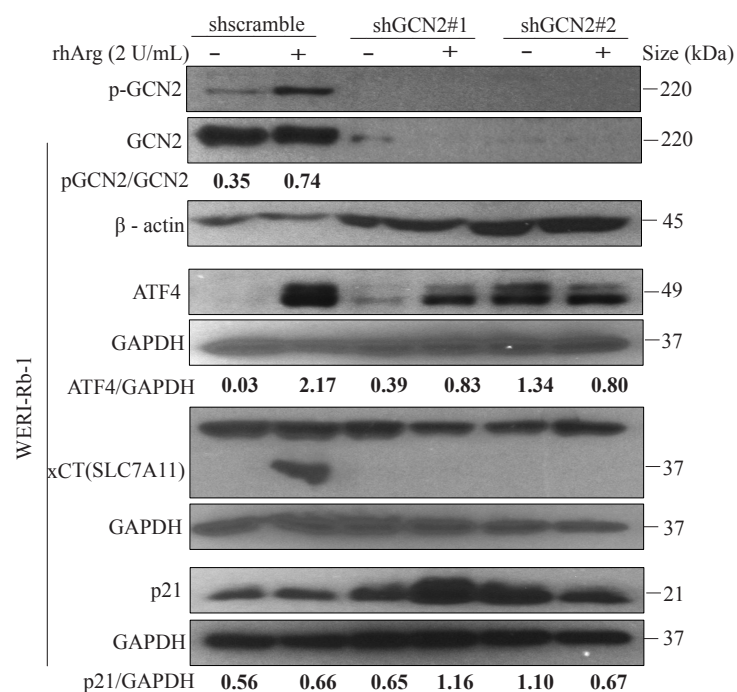

**B**

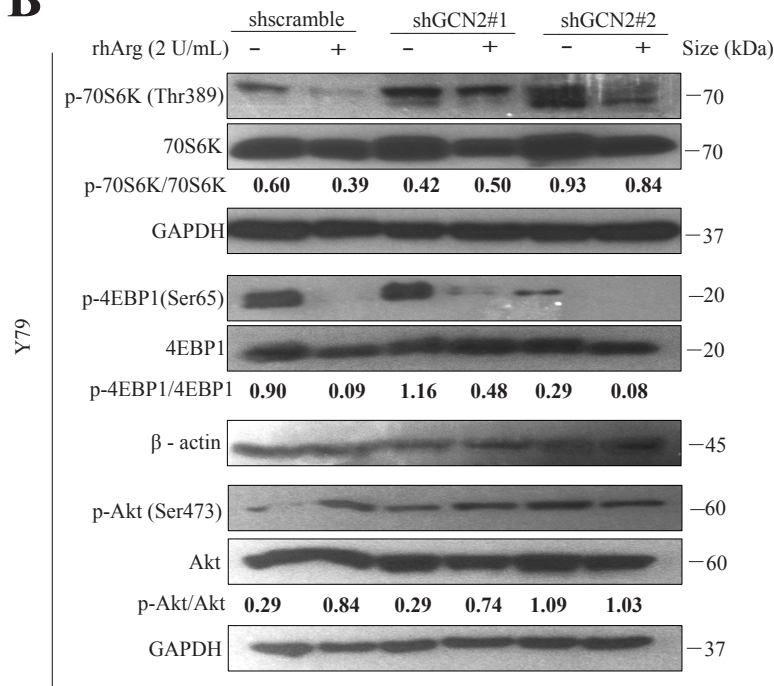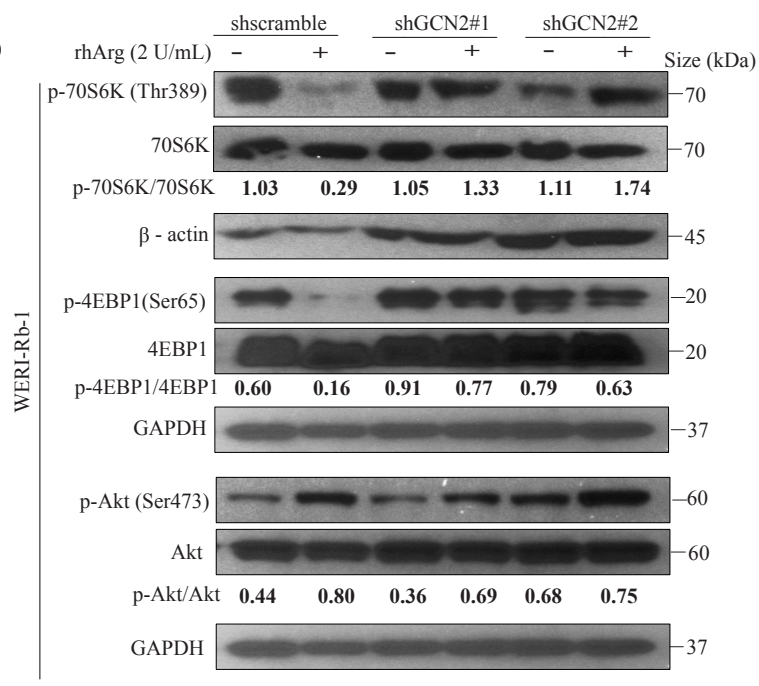

Supplement: Supplementary file 3 — Supplementary Material 3: Supplementary Figure S3. Regulation of GCN2 and mTOR signaling in GCN2-knockdown cells upon arginine deprivation. A. Immunoblot analyses showing the activation of GCN2 signaling in GCN2-knockdown Rb cells upon arginine deprivation. B. Immunoblot analyses showing the inhibition of mTOR signaling in GCN2-knockdown Rb cells upon arginine deprivation. [file 40170_2024_361_MOESM3_ESM.pdf]

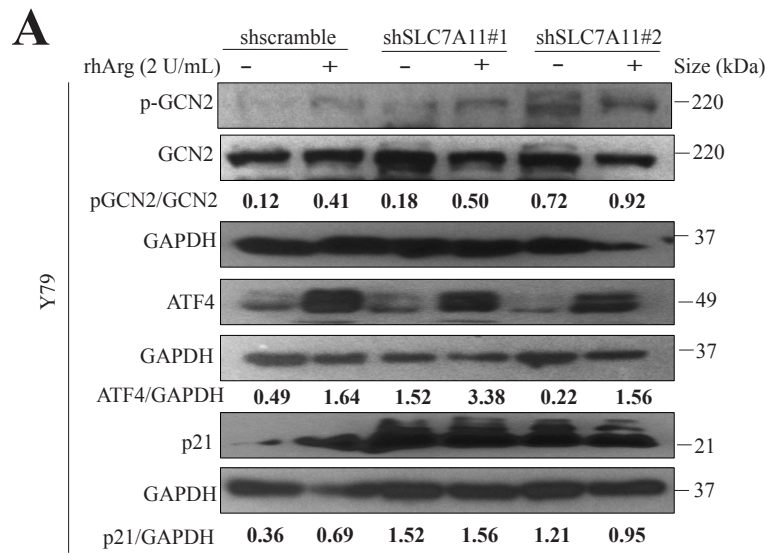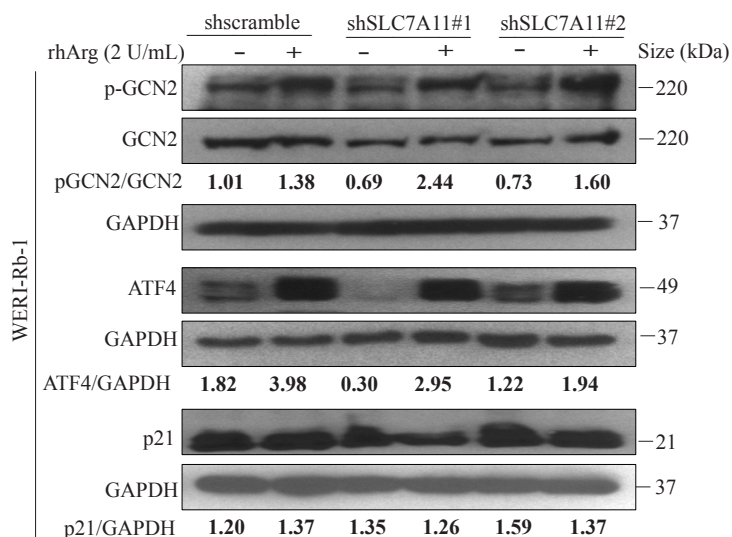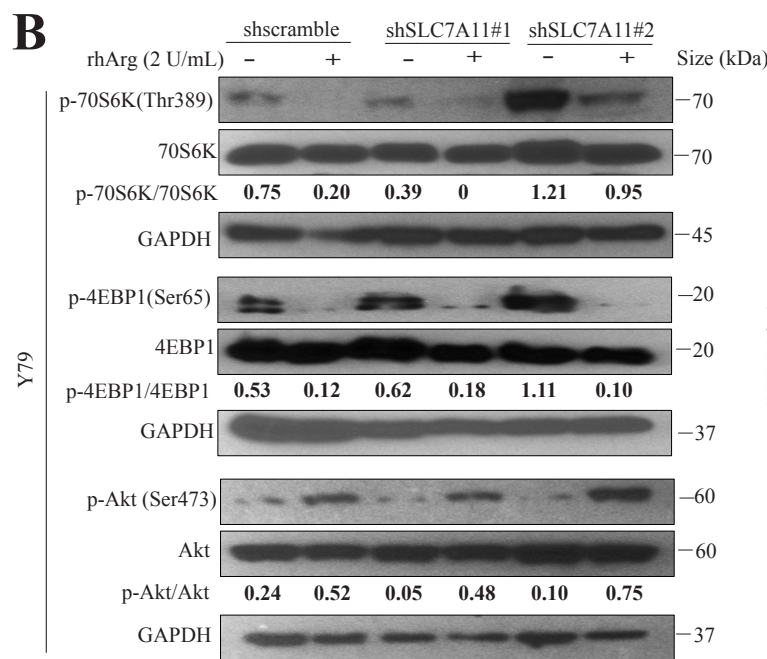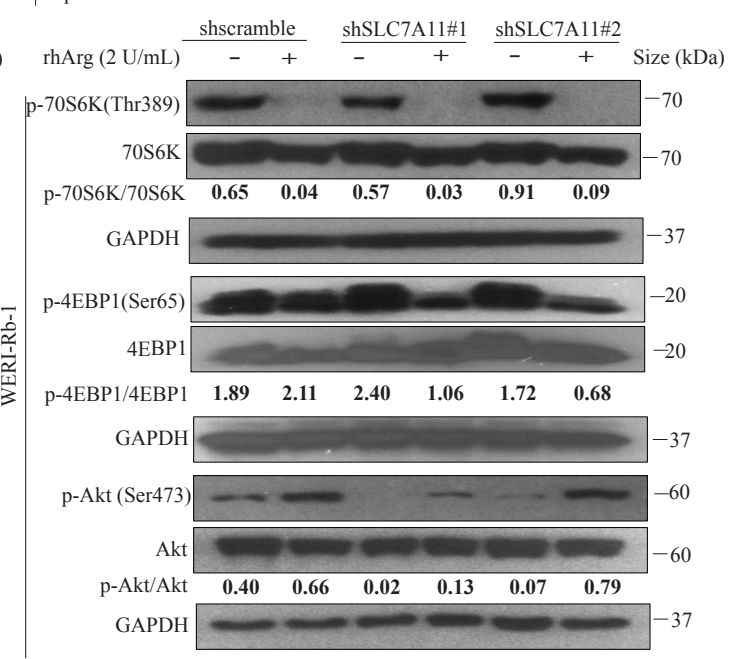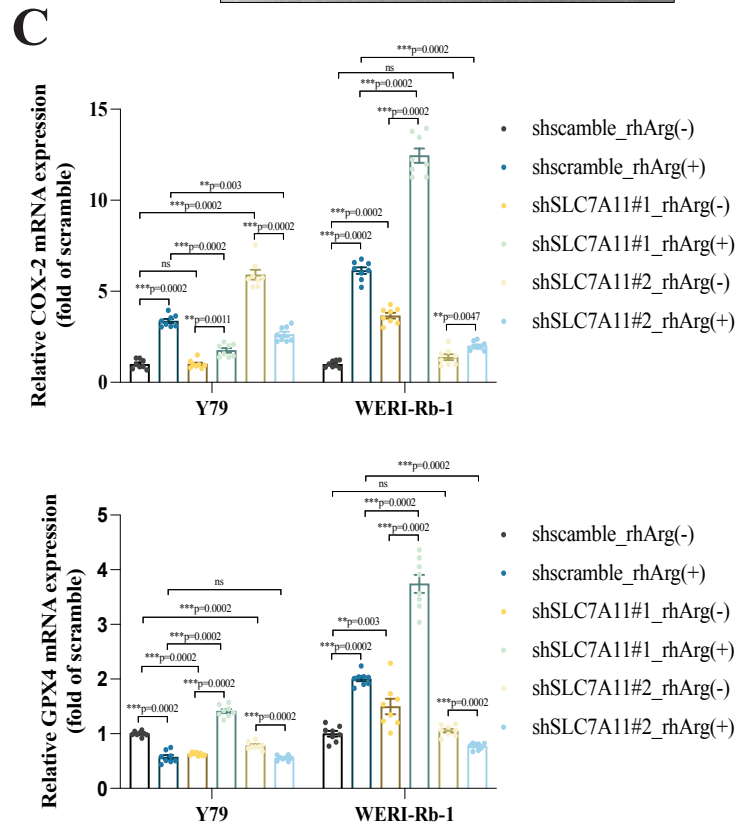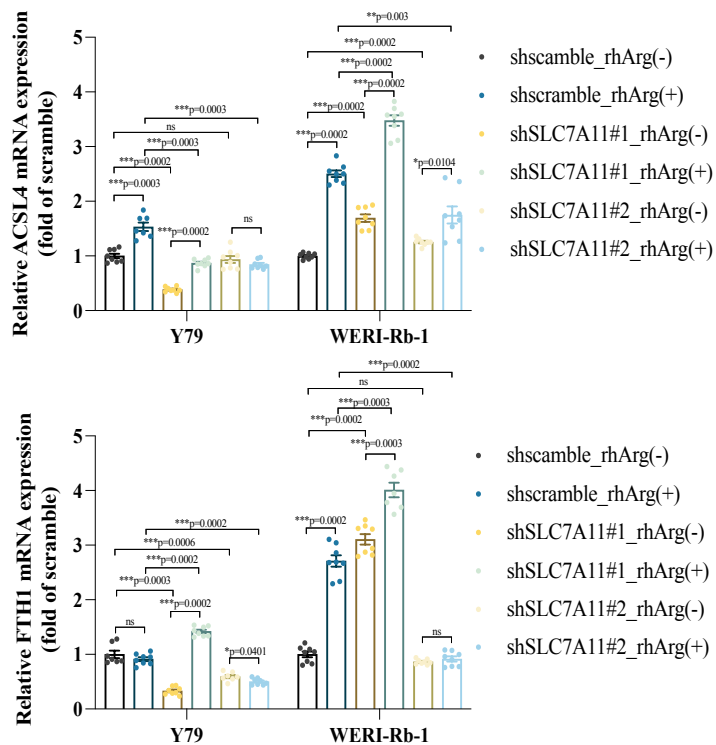

Supplement: Supplementary file 4 — Supplementary Material 4: Supplementary Figure S4. Regulation of GCN2 and mTOR signaling in SLC7A11-knockdown cells upon arginine deprivation. A. Immunoblot analyses showing the activation of GCN2 signaling in SLC7A11-knockdown Rb cells upon arginine deprivation. B. Immunoblot analyses showing the inhibition of mTOR signaling in SLC7A11-knockdown Rb cells upon arginine deprivation. C. q-PCR analysis: COX-2, ACSL4, GPX4, and FTH1 expression levels in SLC7A11-knockdown Y79 and WERI-Rb-1 cells upon arginine deprivation. [file 40170_2024_361_MOESM4_ESM.pdf]

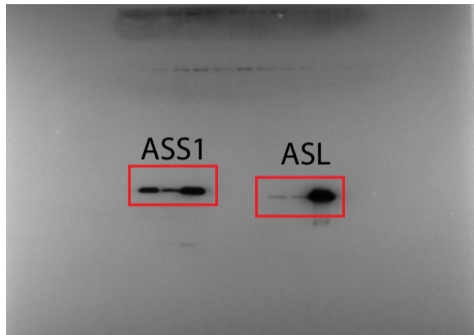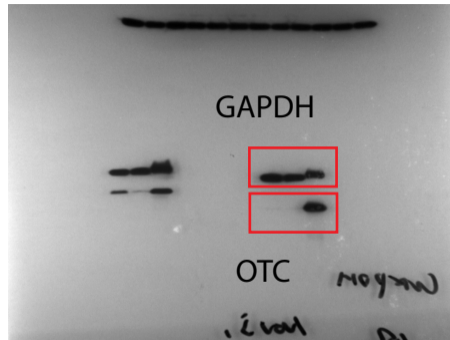

Supplement: Supplementary file 5 — Supplementary Material 5. [file 40170_2024_361_MOESM5_ESM.zip › Fig 1D_raw.pdf]

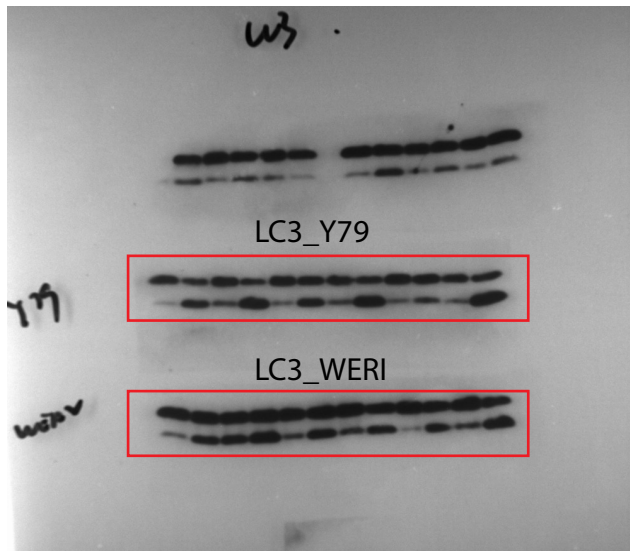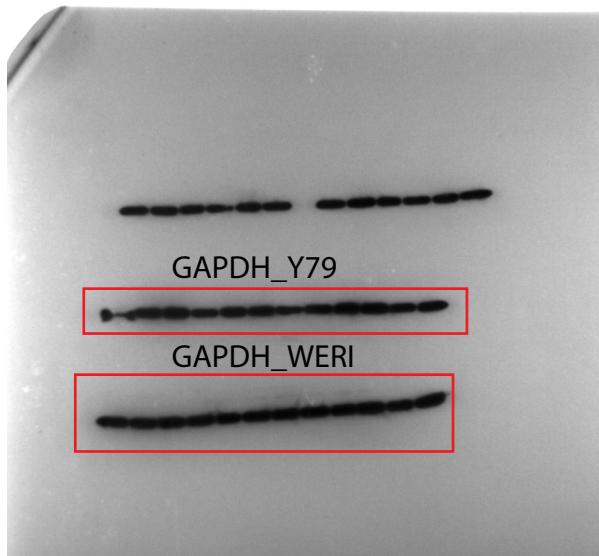

Supplement: Supplementary file 5 — Supplementary Material 5. [file 40170_2024_361_MOESM5_ESM.zip › Fig 2B_raw.pdf]

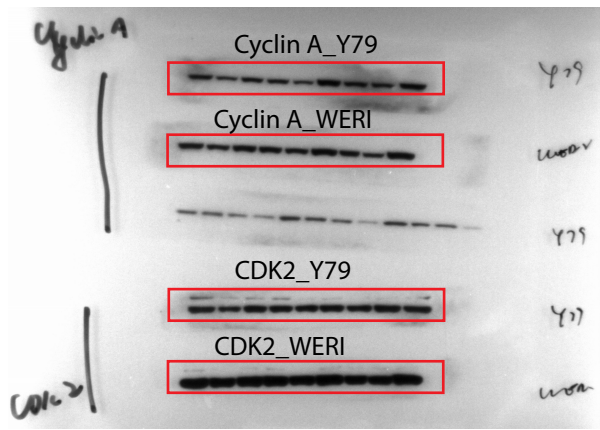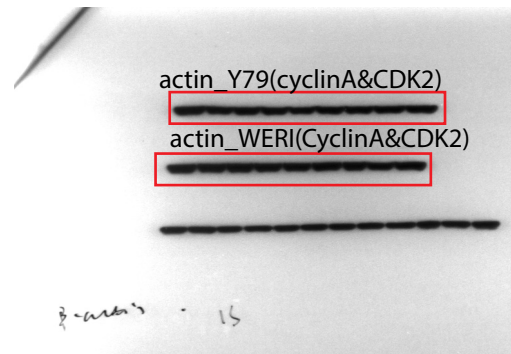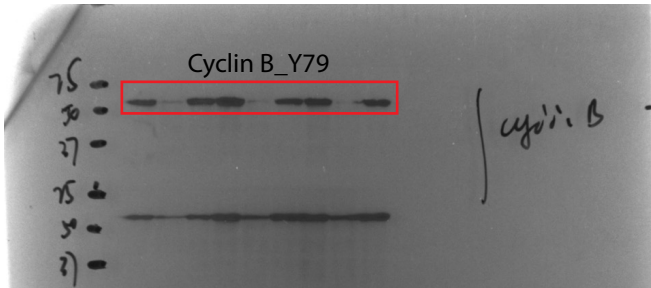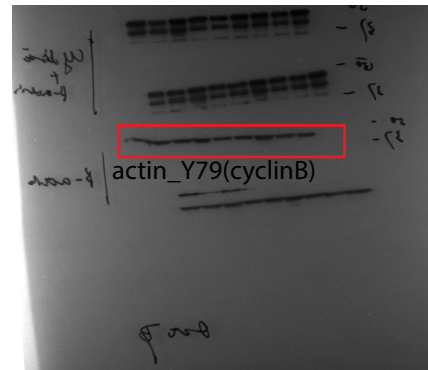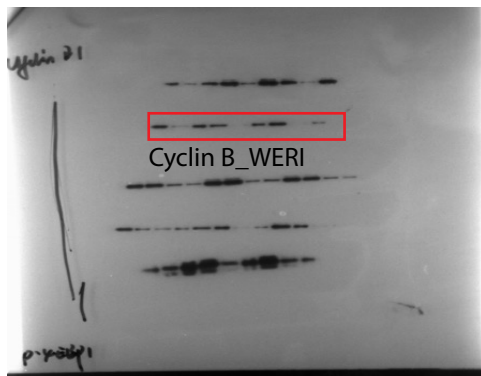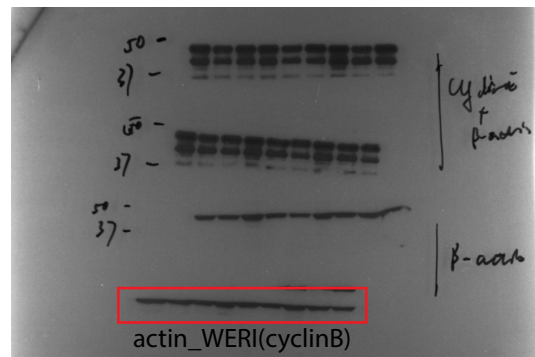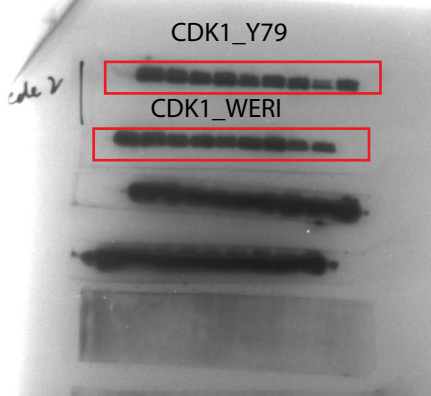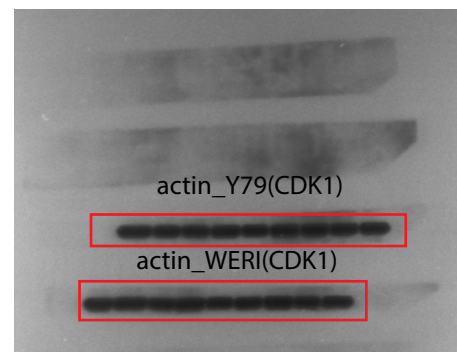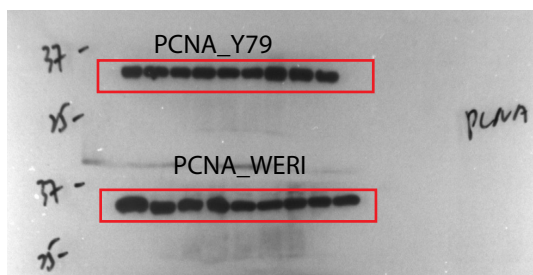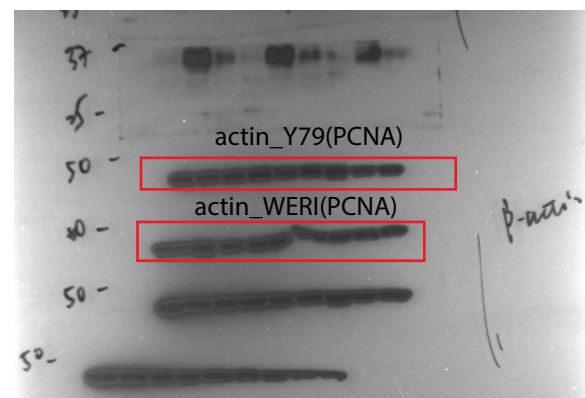

Supplement: Supplementary file 5 — Supplementary Material 5. [file 40170_2024_361_MOESM5_ESM.zip › Fig 2D_raw.pdf]

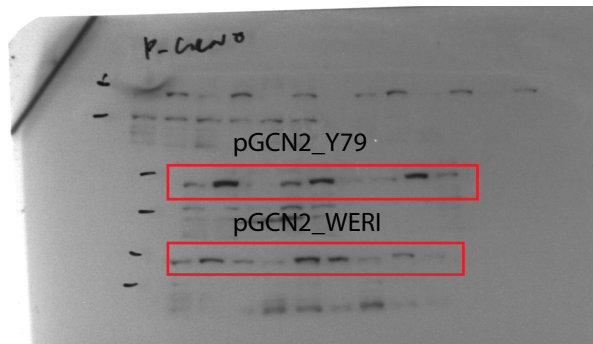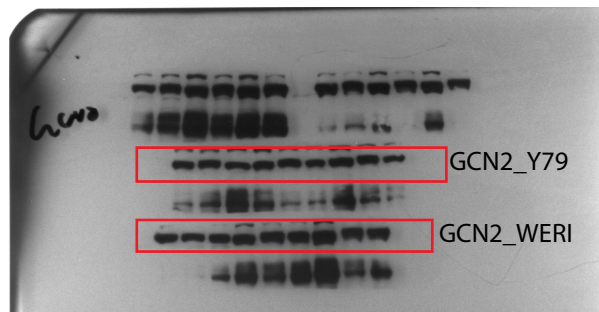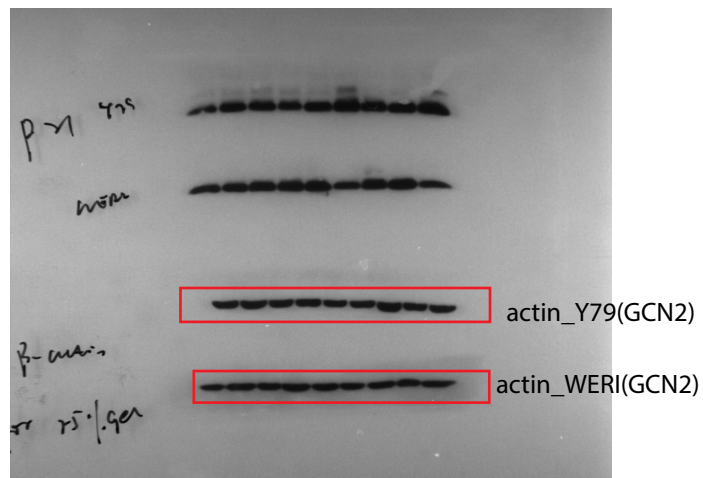

Supplement: Supplementary file 5 — Supplementary Material 5. [file 40170_2024_361_MOESM5_ESM.zip › Fig 4A_raw.pdf]

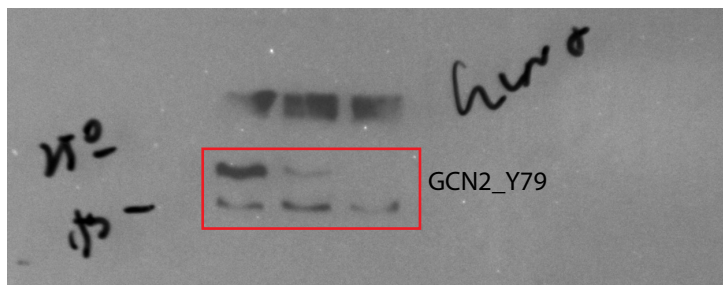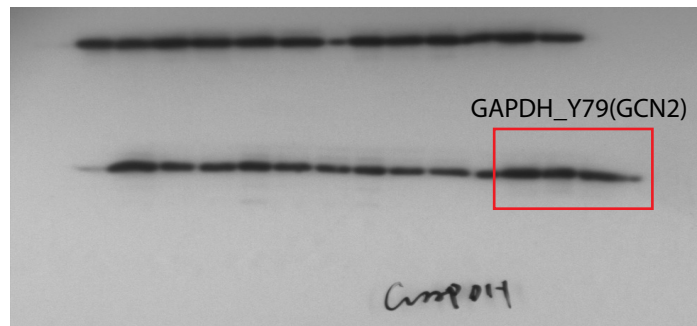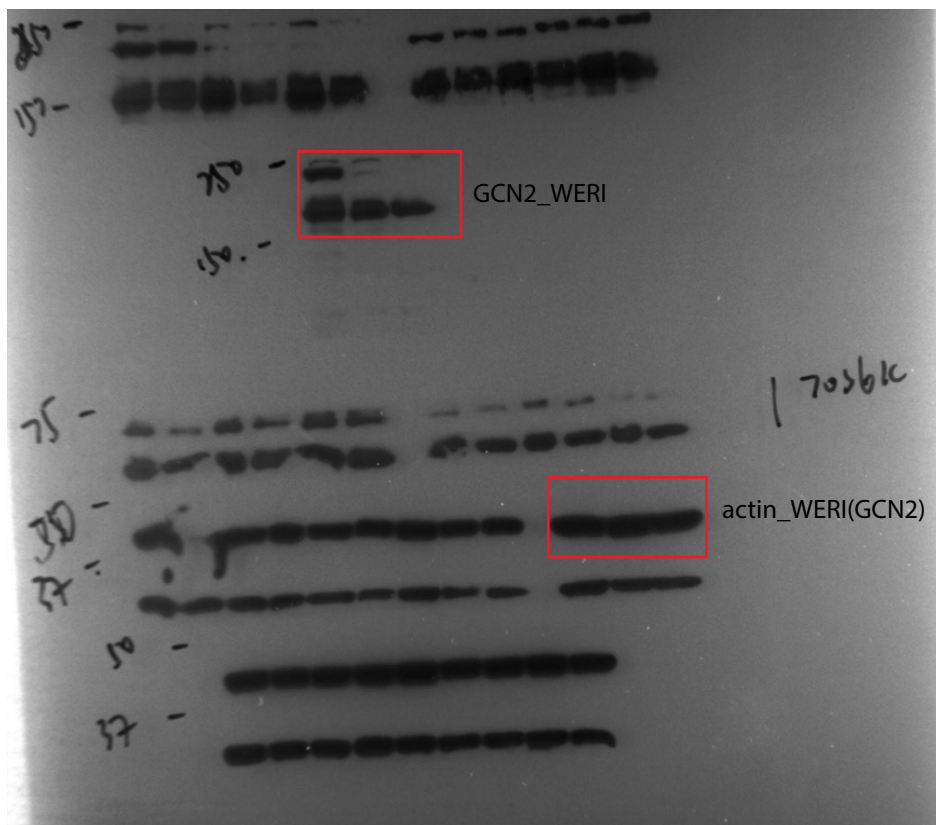

Supplement: Supplementary file 5 — Supplementary Material 5. [file 40170_2024_361_MOESM5_ESM.zip › Fig 4B_raw.pdf]

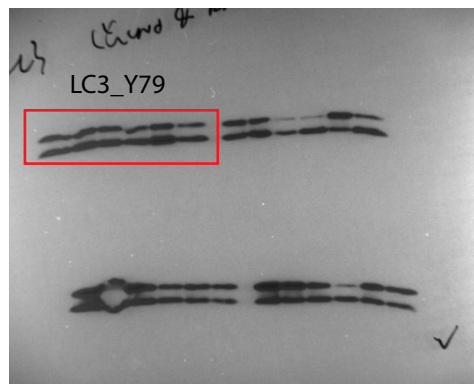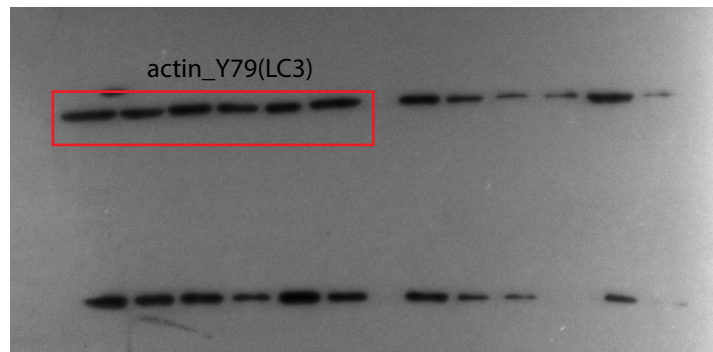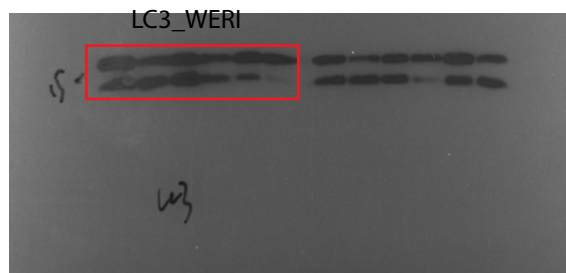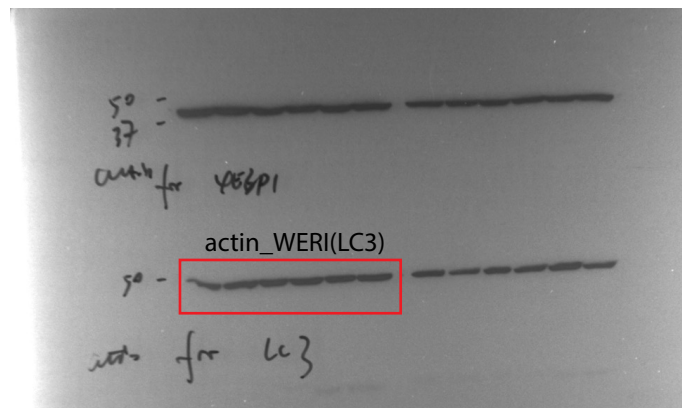

Supplement: Supplementary file 5 — Supplementary Material 5. [file 40170_2024_361_MOESM5_ESM.zip › Fig 4E_raw.pdf]

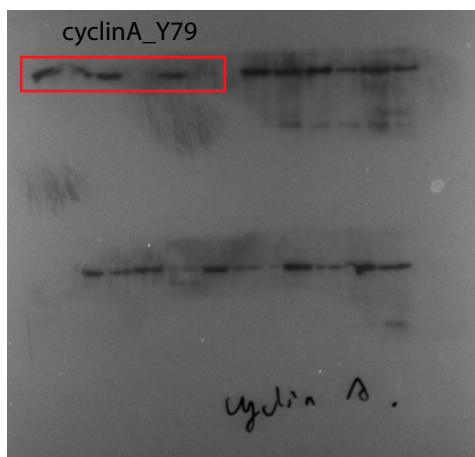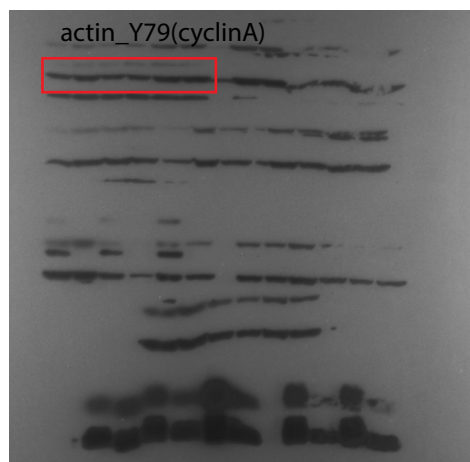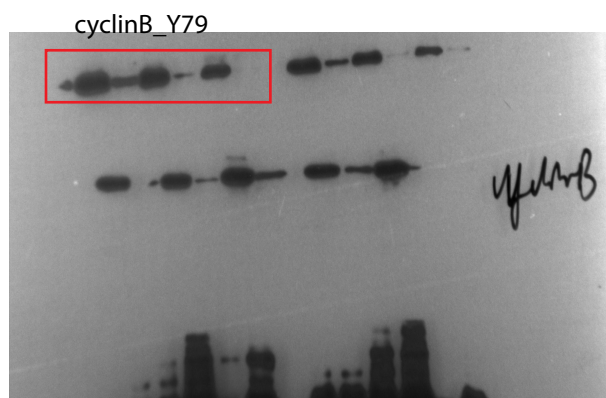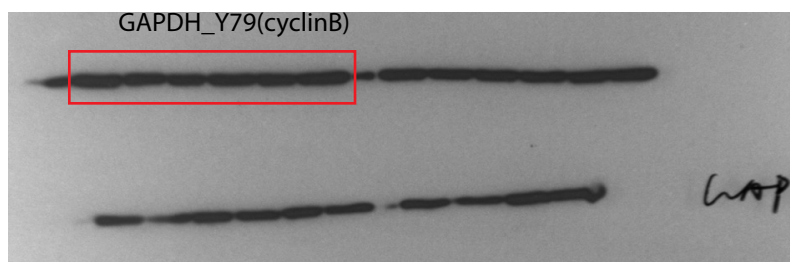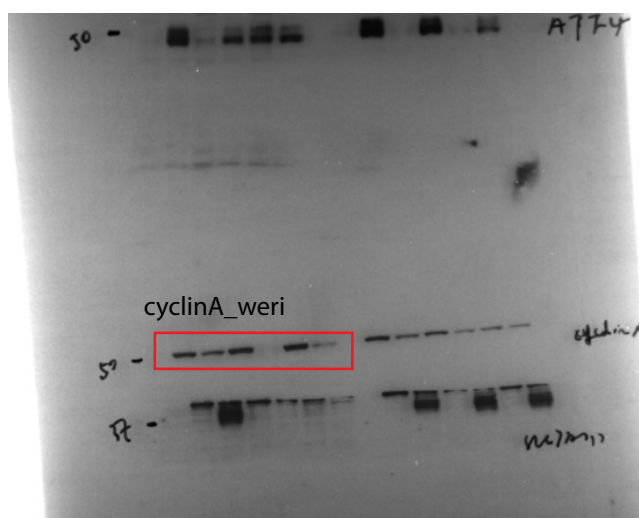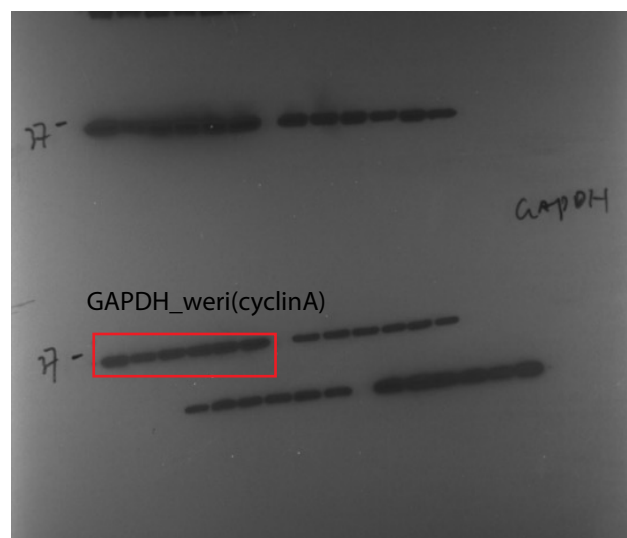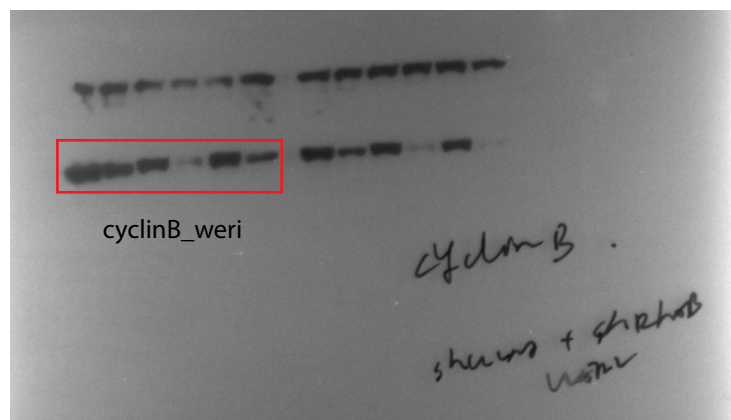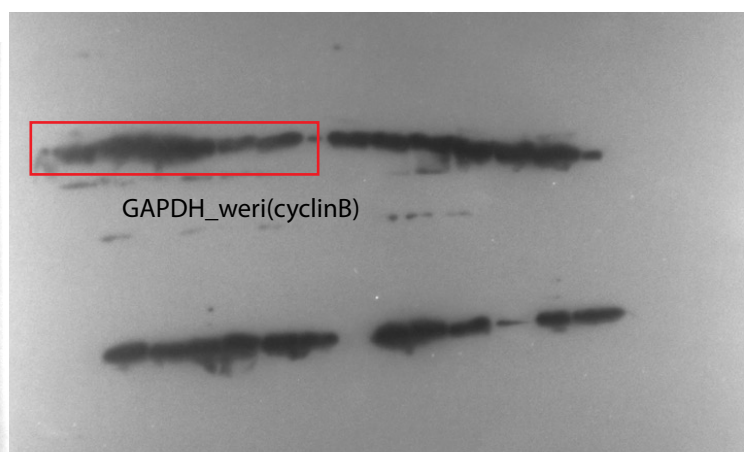

Supplement: Supplementary file 5 — Supplementary Material 5. [file 40170_2024_361_MOESM5_ESM.zip › Fig 4F_raw.pdf]

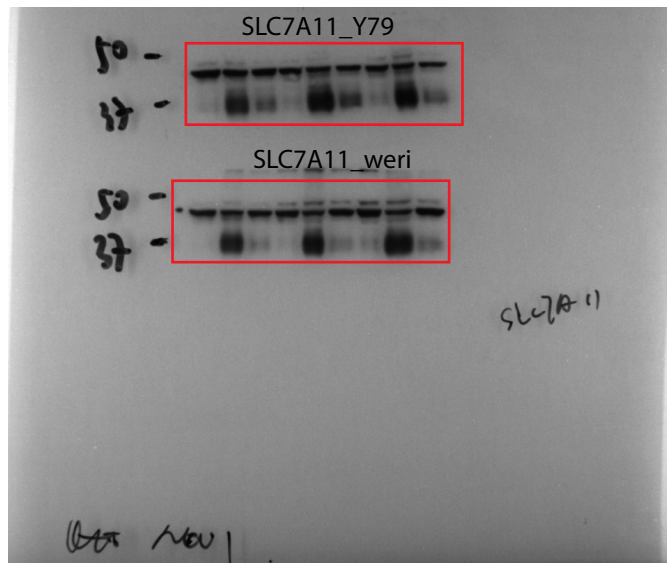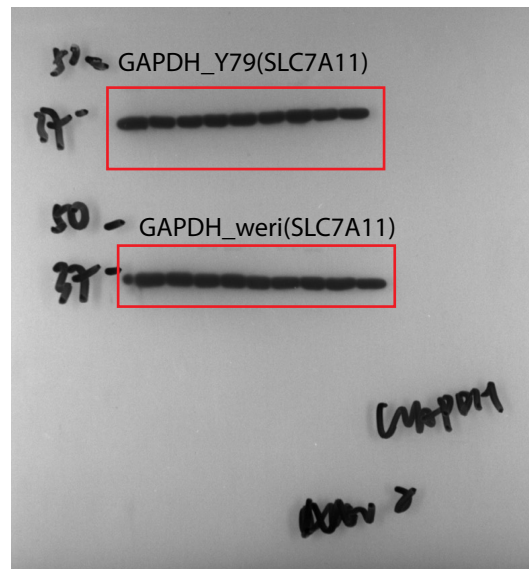

Supplement: Supplementary file 5 — Supplementary Material 5. [file 40170_2024_361_MOESM5_ESM.zip › Fig 5A_raw.pdf]

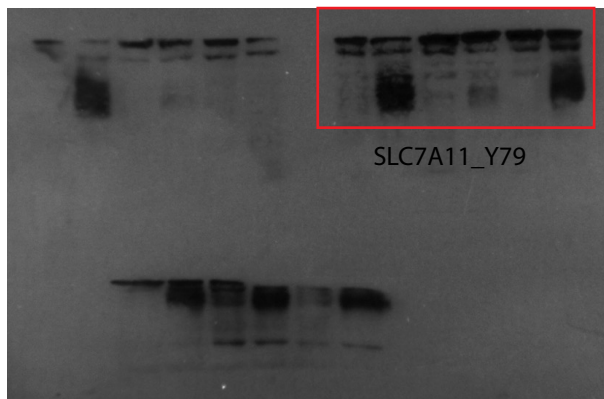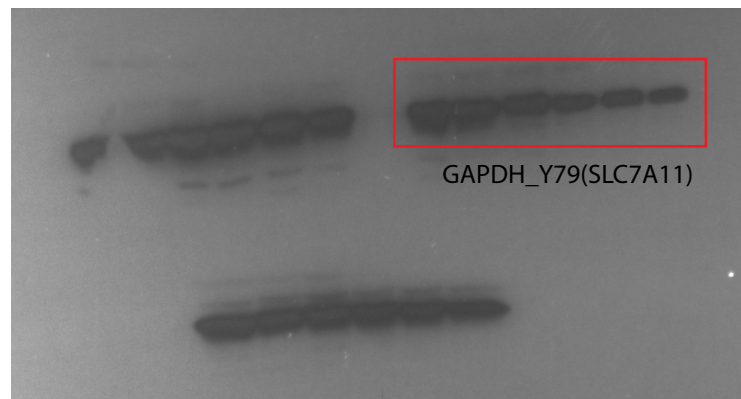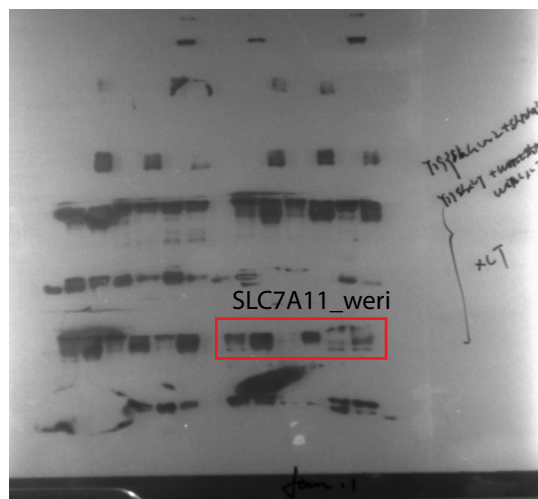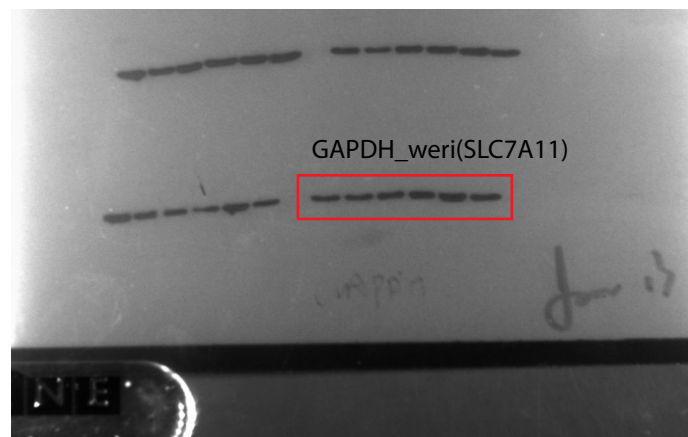

Supplement: Supplementary file 5 — Supplementary Material 5. [file 40170_2024_361_MOESM5_ESM.zip › Fig 5B_raw.pdf]

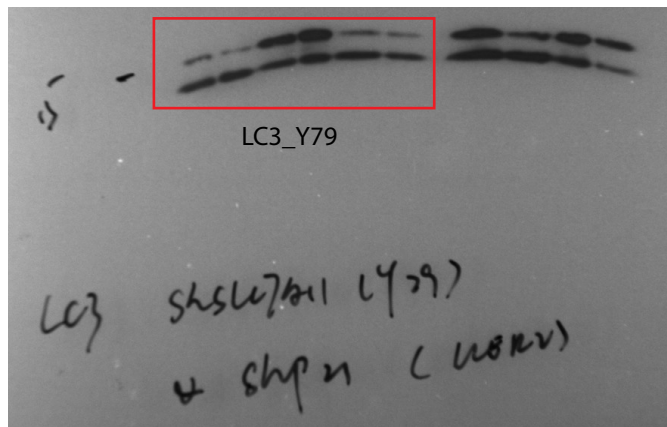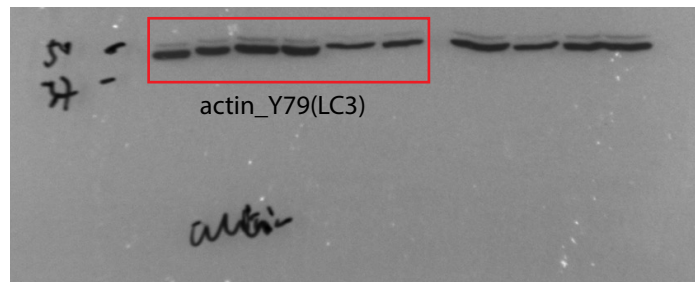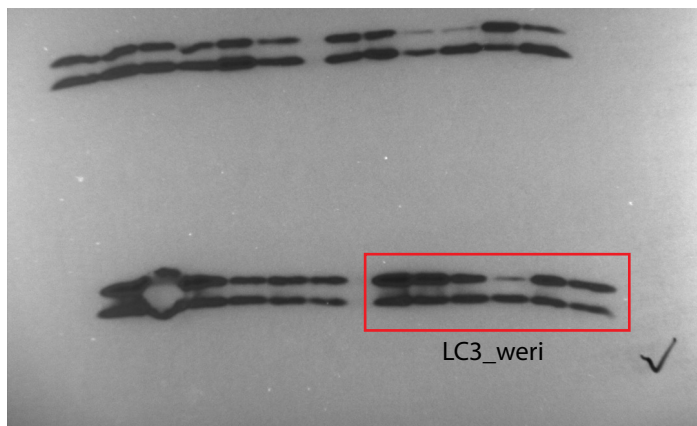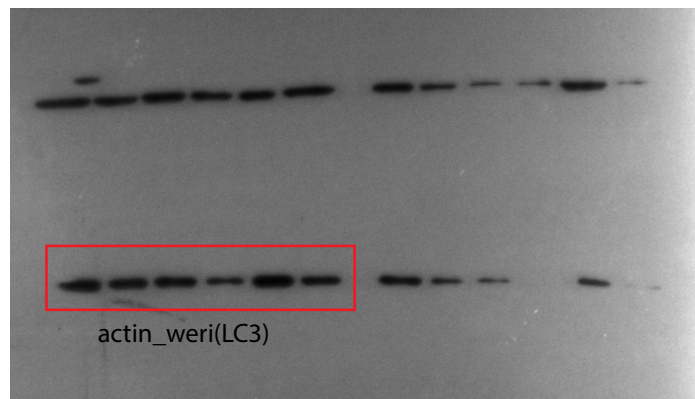

Supplement: Supplementary file 5 — Supplementary Material 5. [file 40170_2024_361_MOESM5_ESM.zip › Fig 5E_raw.pdf]

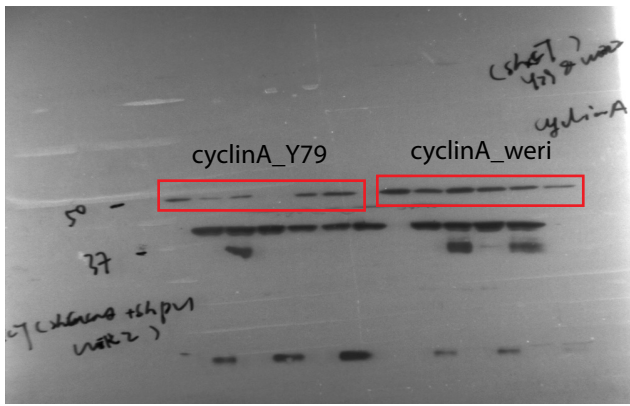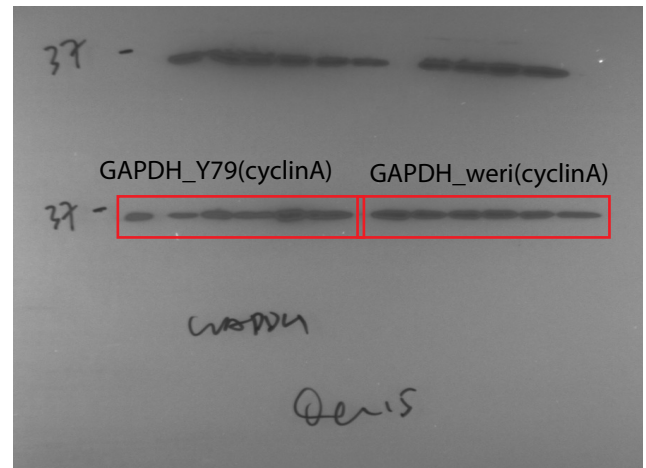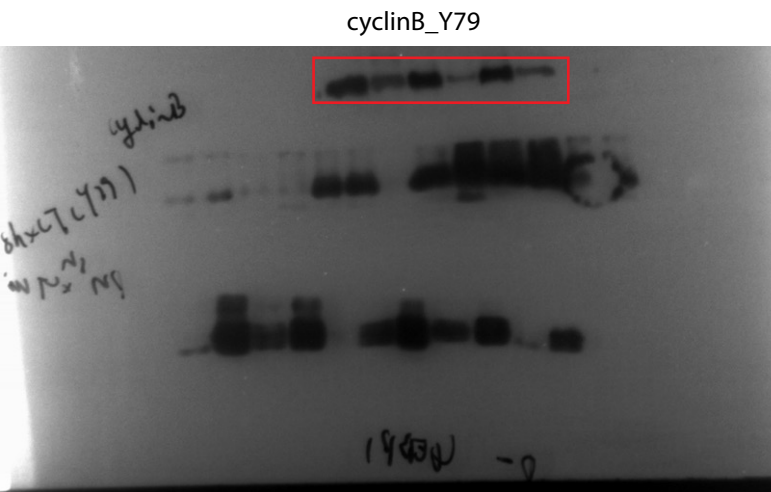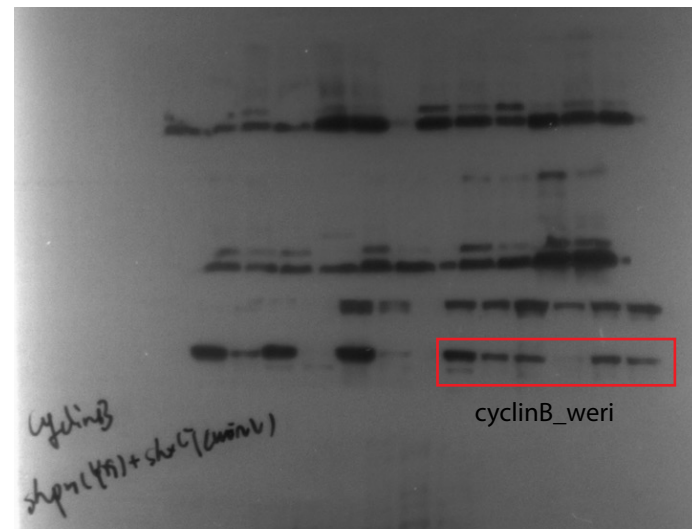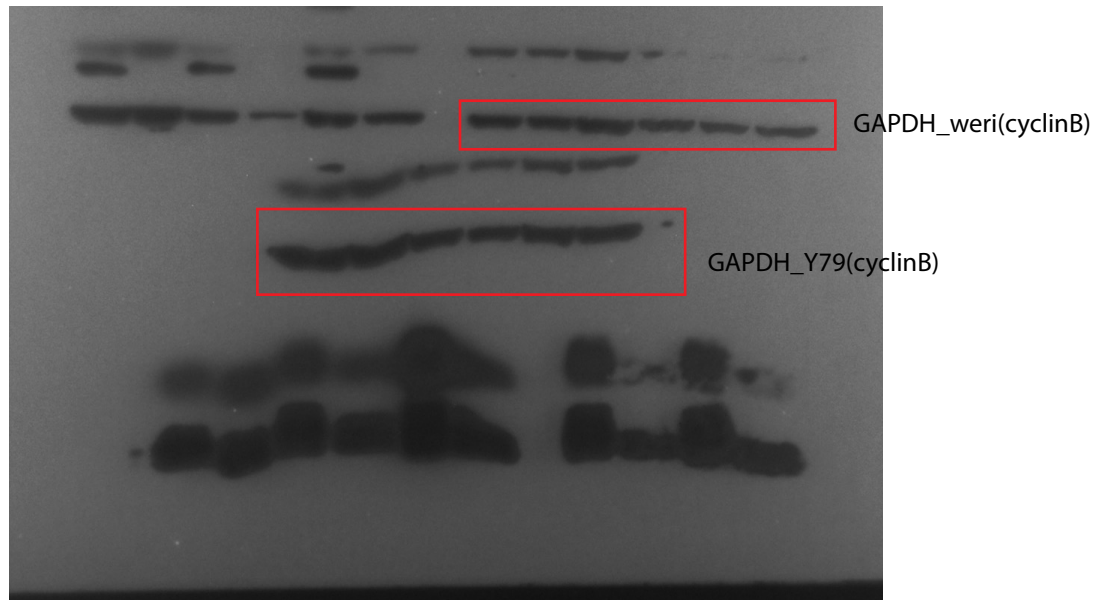

Supplement: Supplementary file 5 — Supplementary Material 5. [file 40170_2024_361_MOESM5_ESM.zip › Fig 5F_raw.pdf]

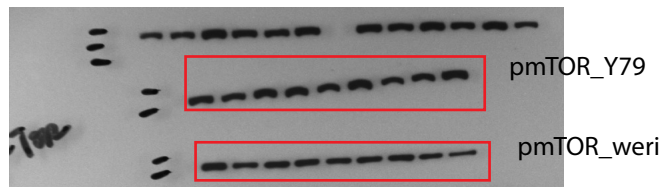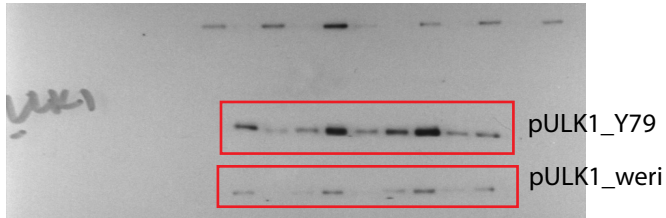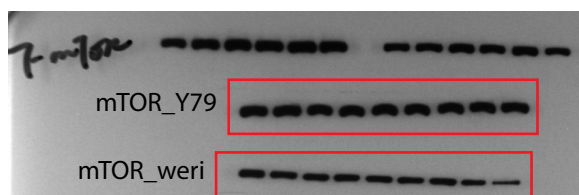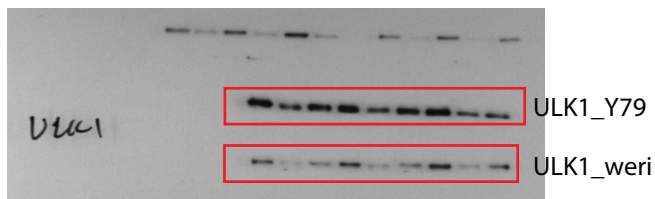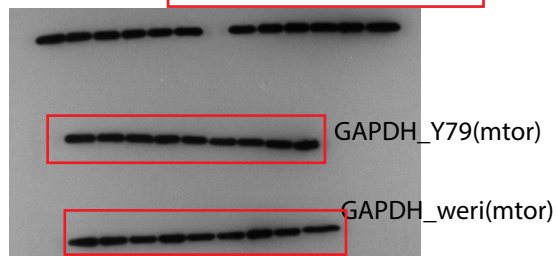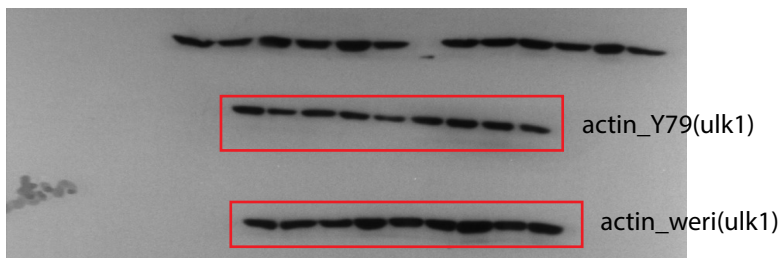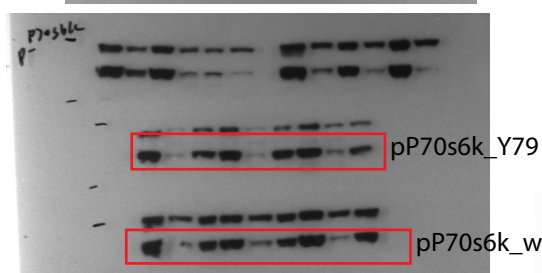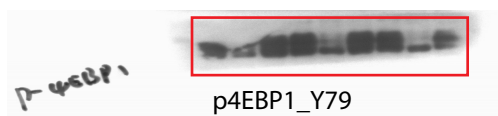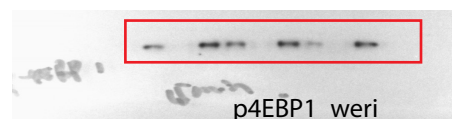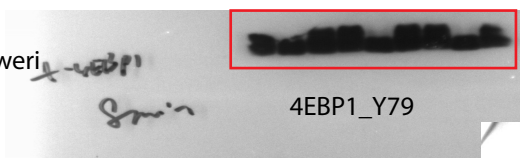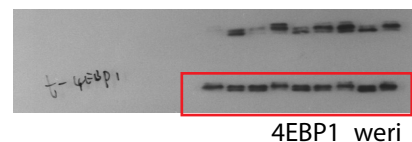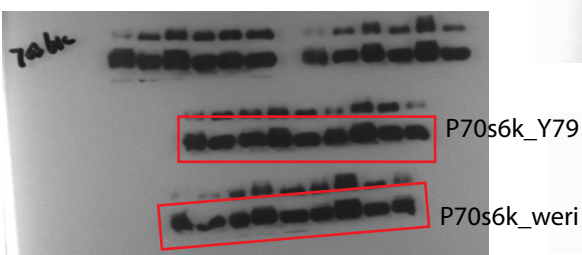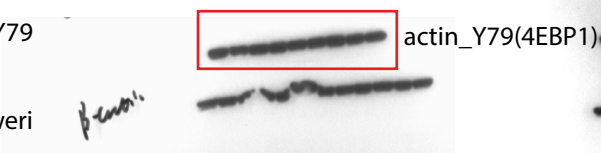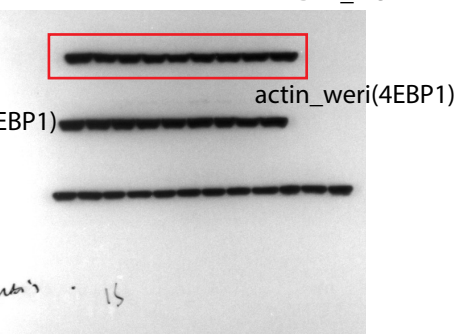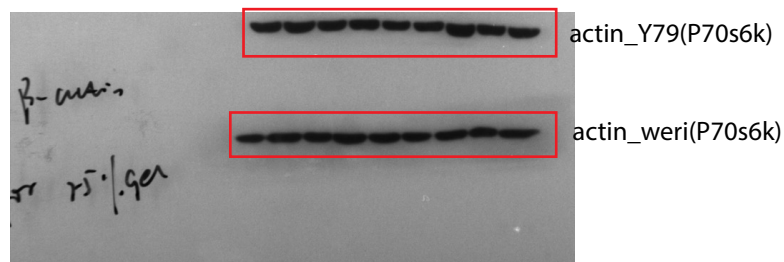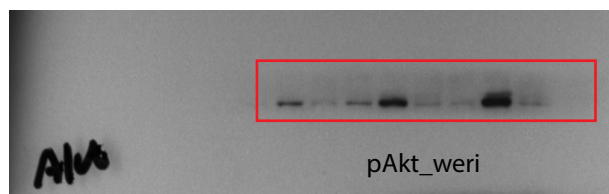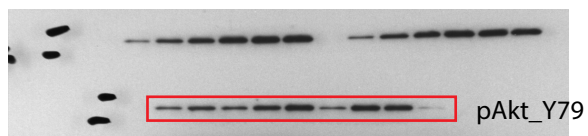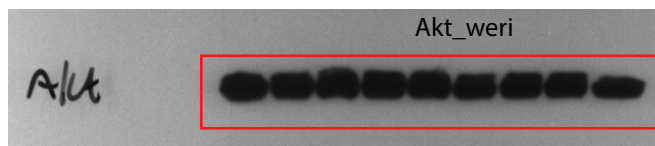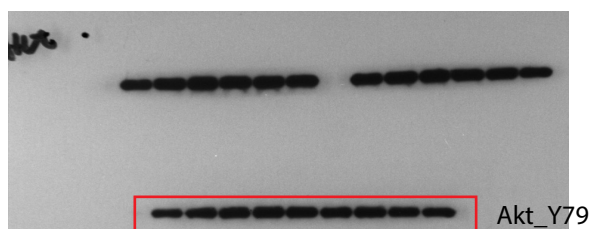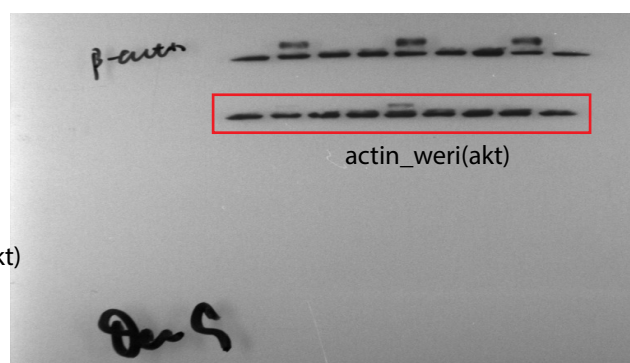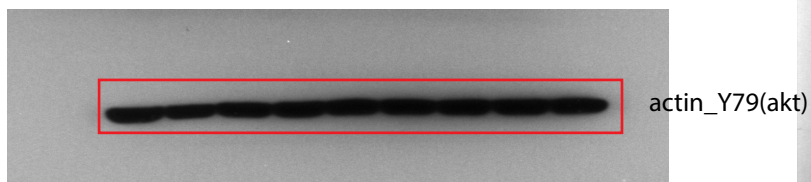

Supplement: Supplementary file 5 — Supplementary Material 5. [file 40170_2024_361_MOESM5_ESM.zip › Fig Suppl Fig2A_raw.pdf]

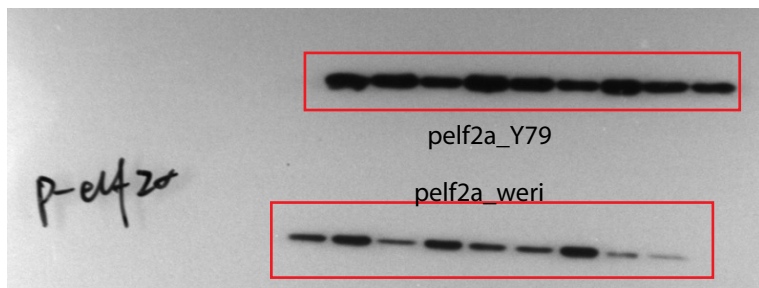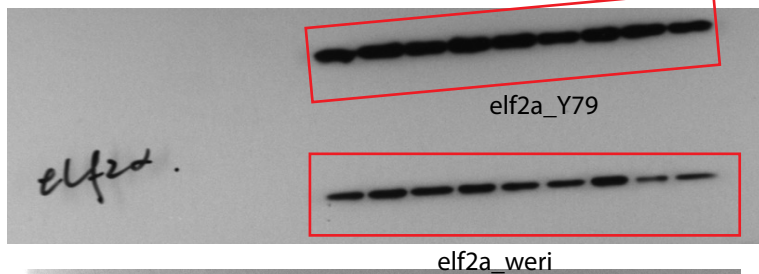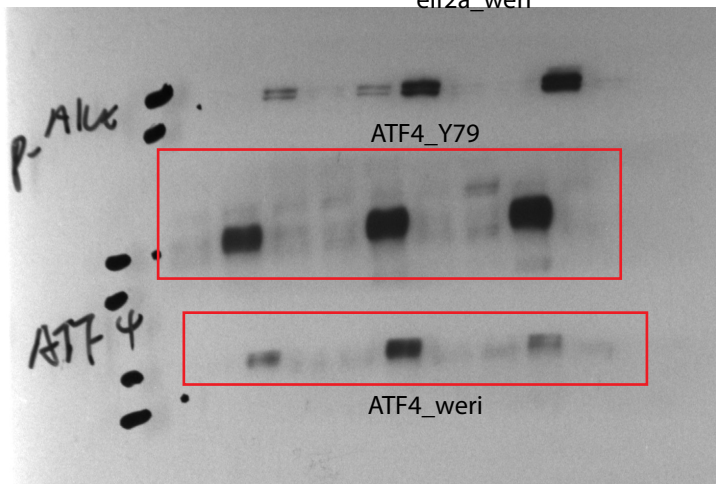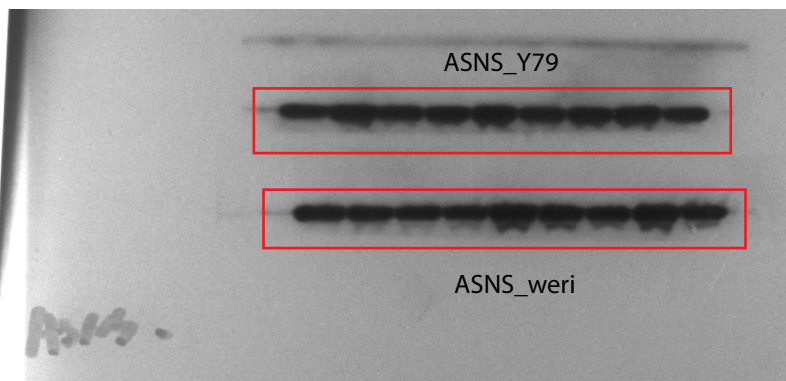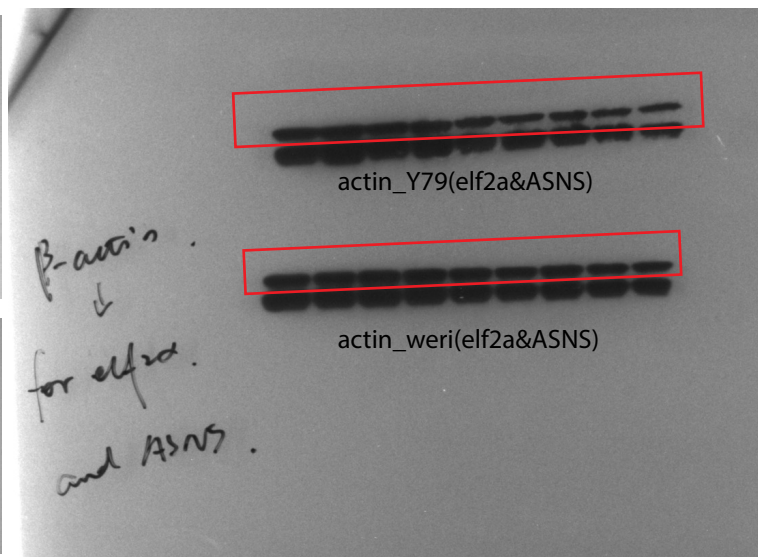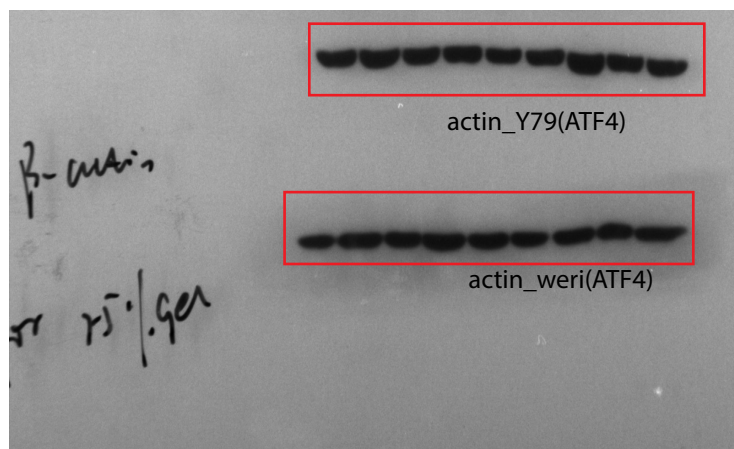

Supplement: Supplementary file 5 — Supplementary Material 5. [file 40170_2024_361_MOESM5_ESM.zip › Fig Suppl Fig2B_raw.pdf]

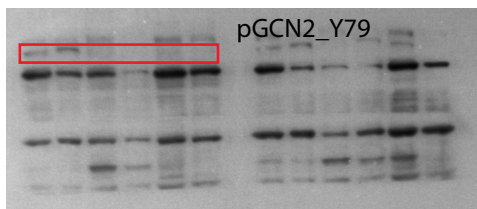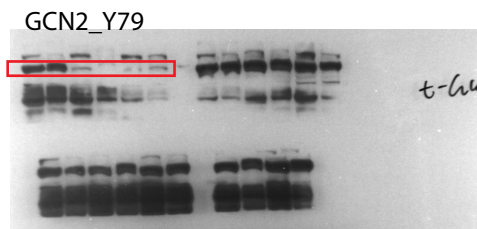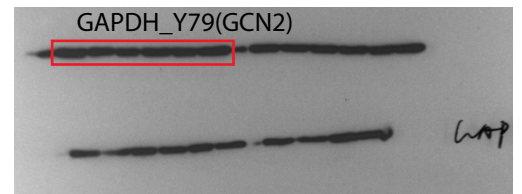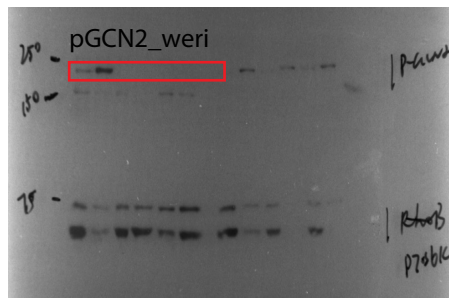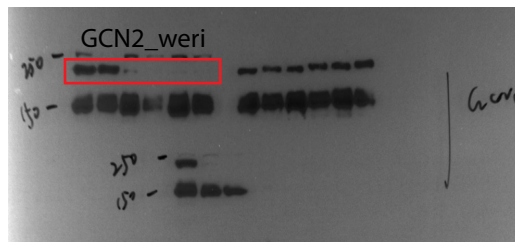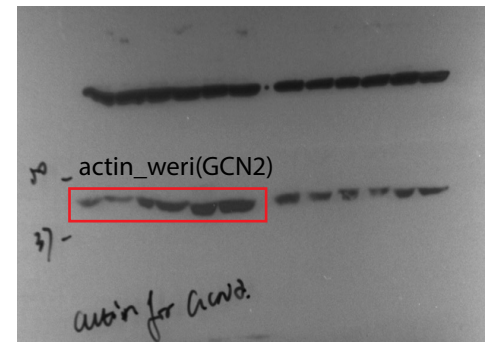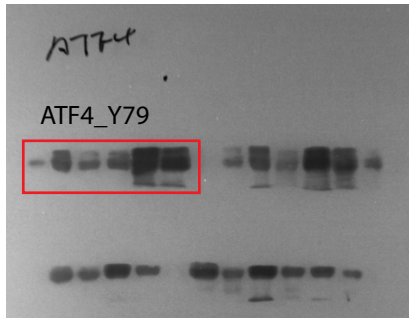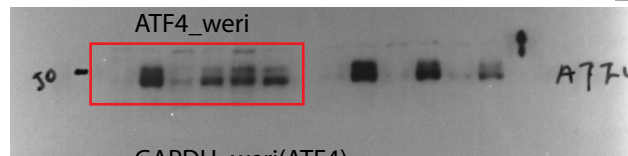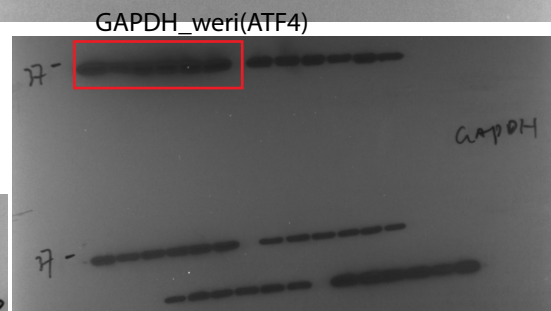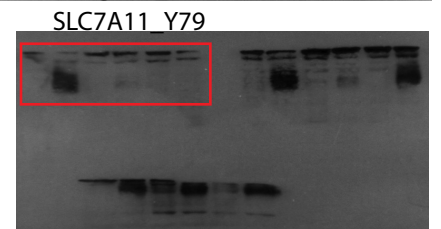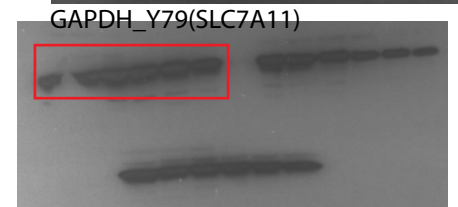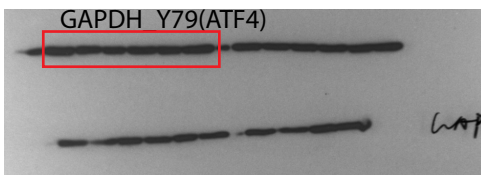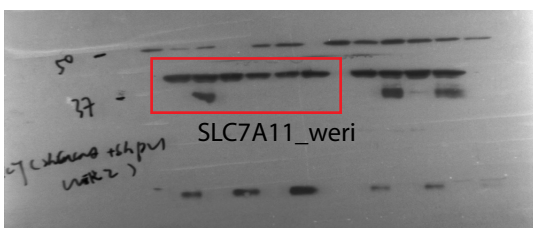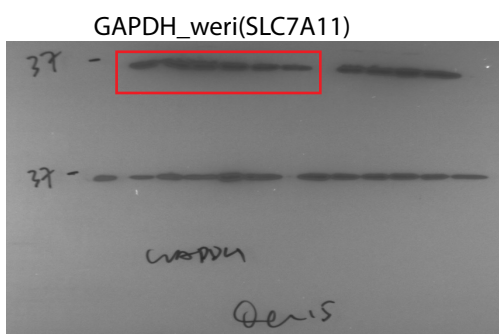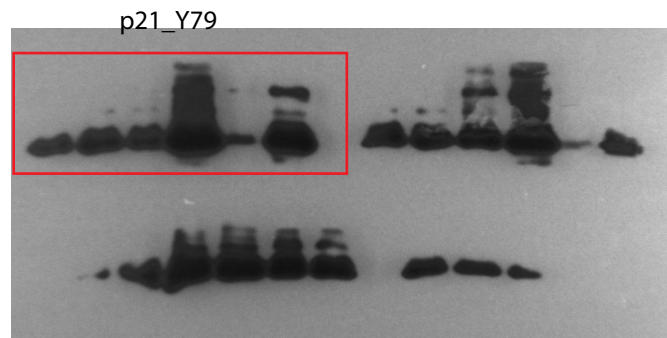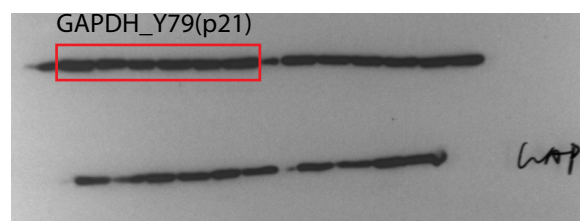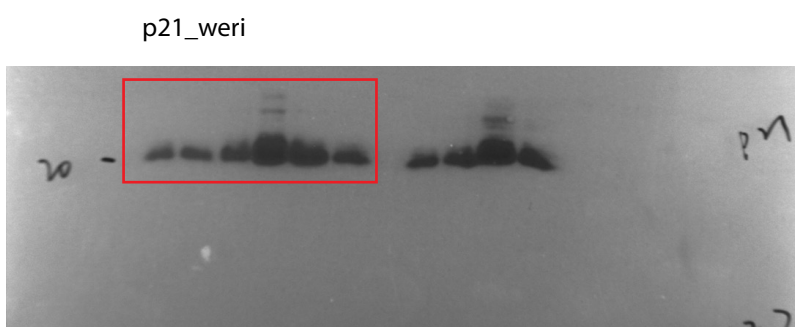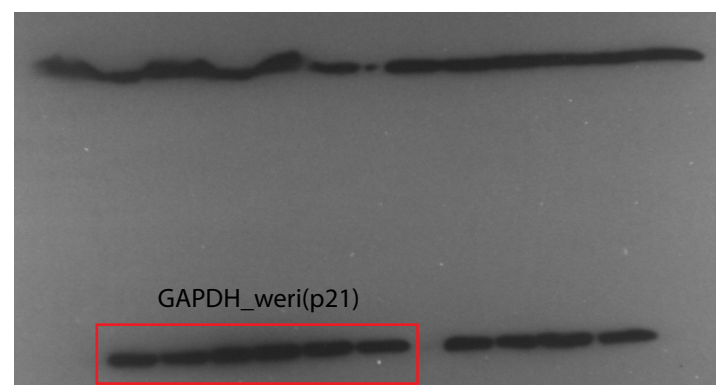

Supplement: Supplementary file 5 — Supplementary Material 5. [file 40170_2024_361_MOESM5_ESM.zip › Fig Suppl Fig3A_raw.pdf]

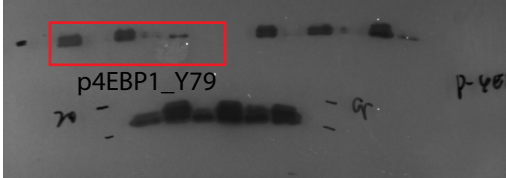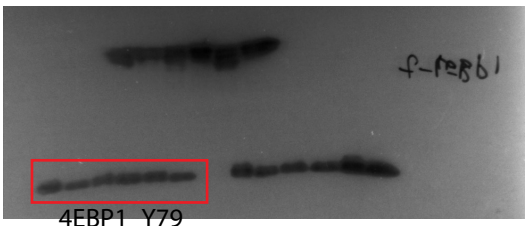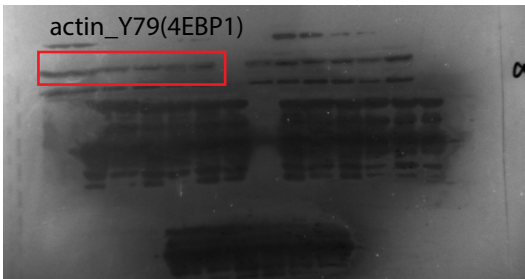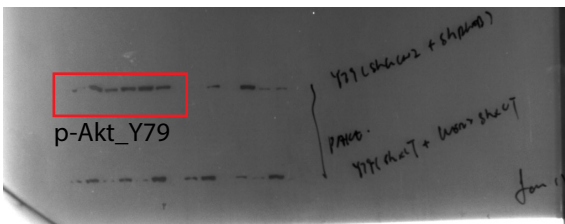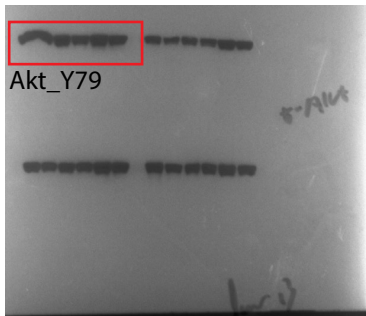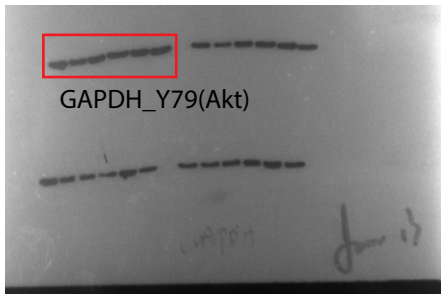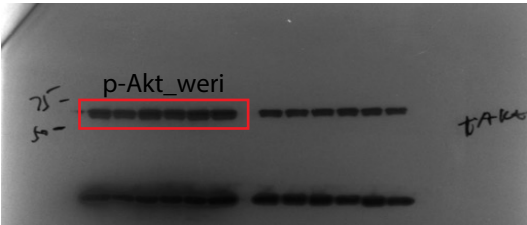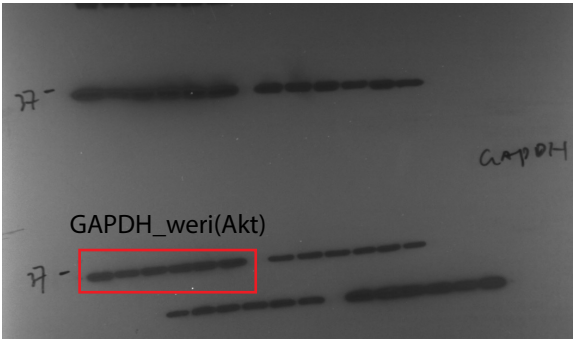

Supplement: Supplementary file 5 — Supplementary Material 5. [file 40170_2024_361_MOESM5_ESM.zip › Fig Suppl Fig3B_raw.pdf]

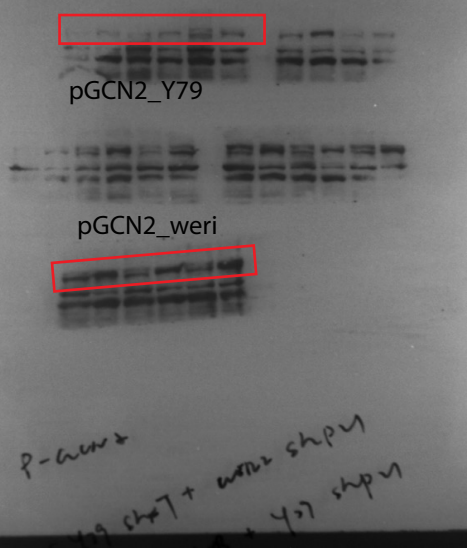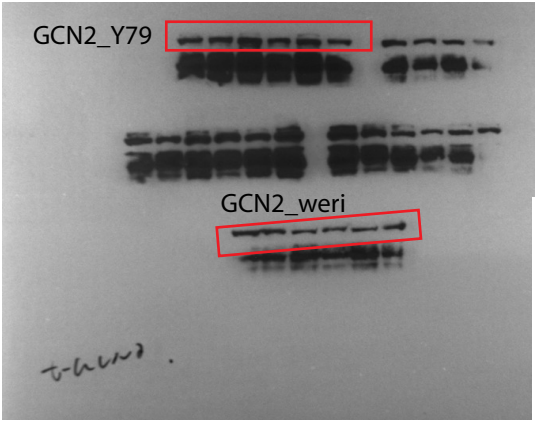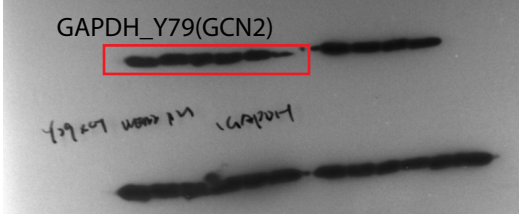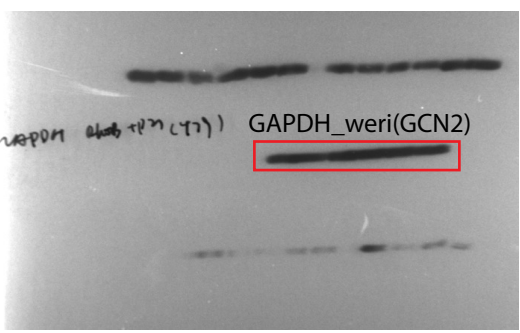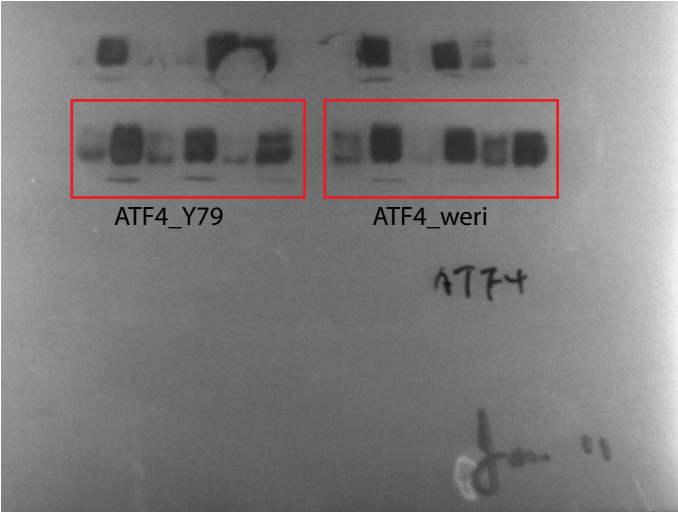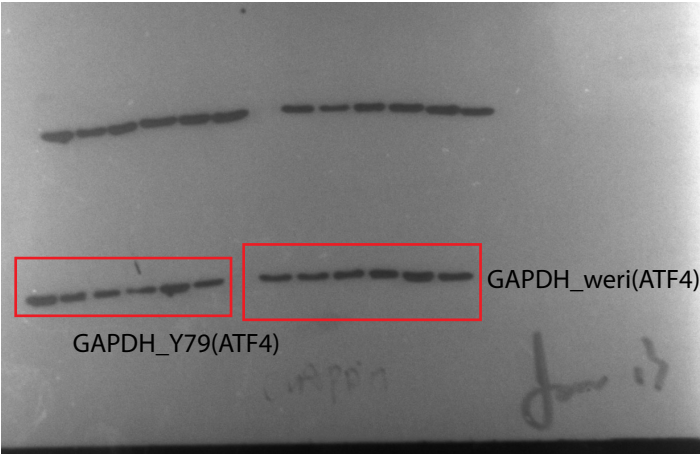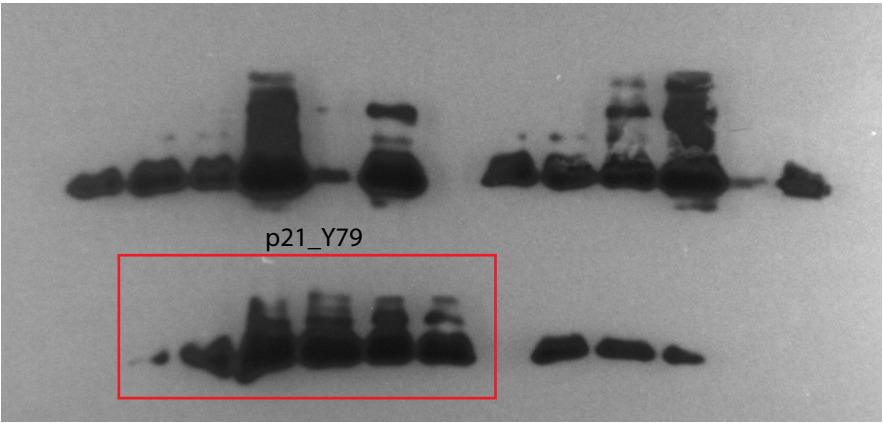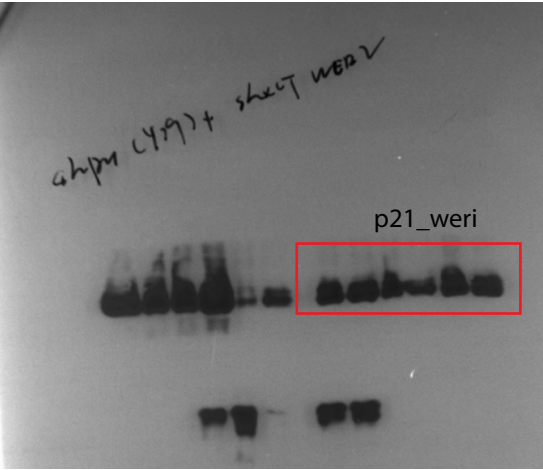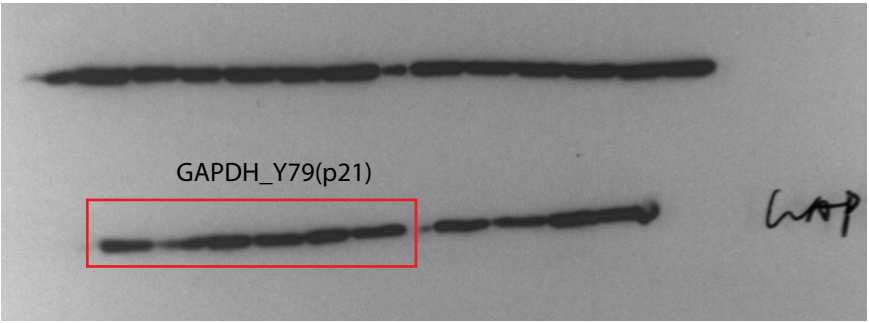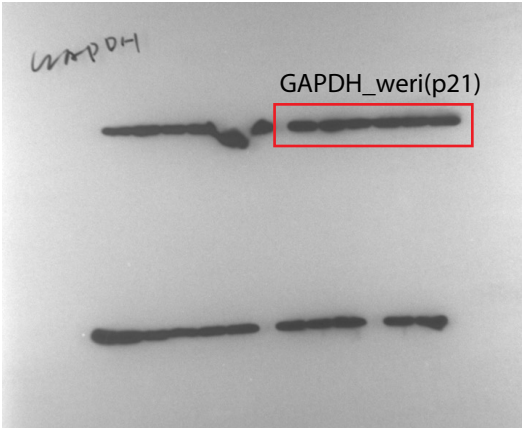

Supplement: Supplementary file 5 — Supplementary Material 5. [file 40170_2024_361_MOESM5_ESM.zip › Fig Suppl Fig4A_raw.pdf]

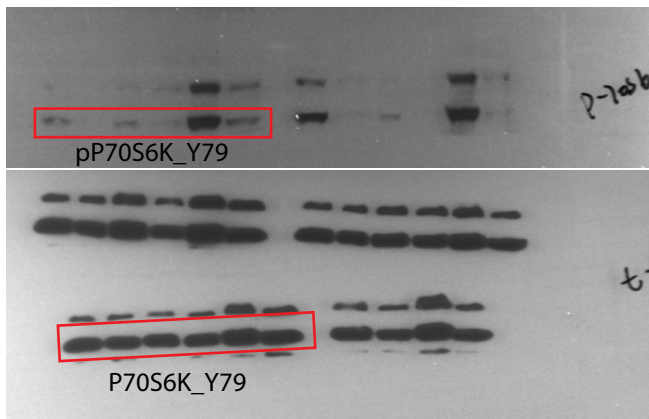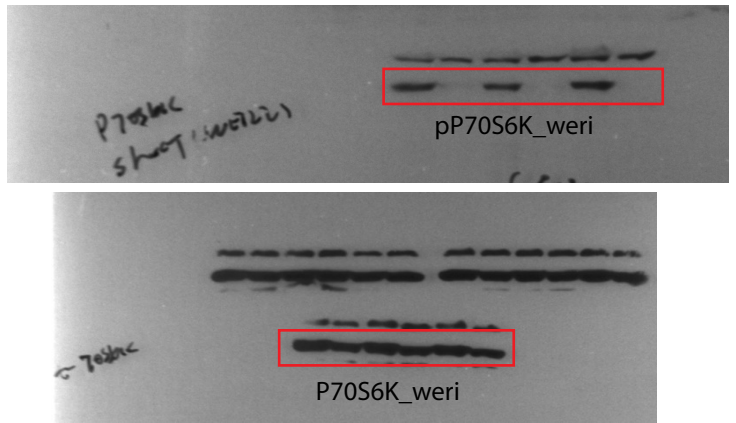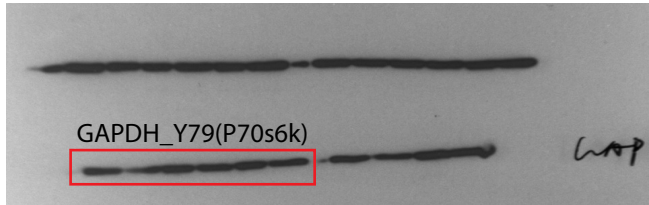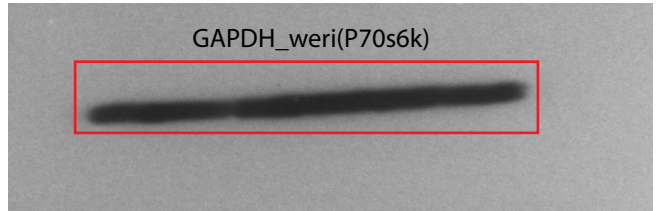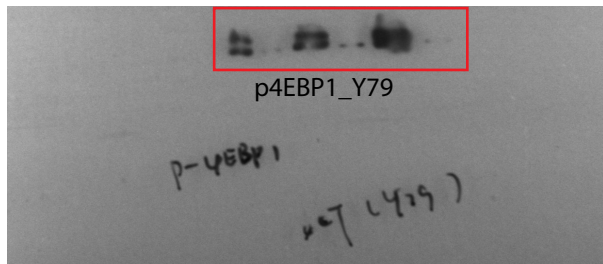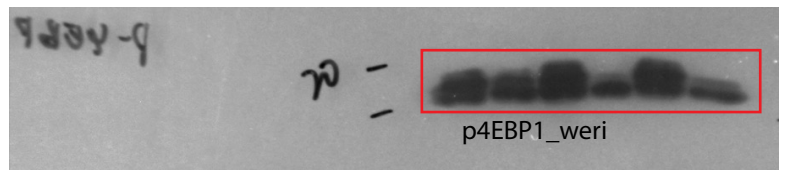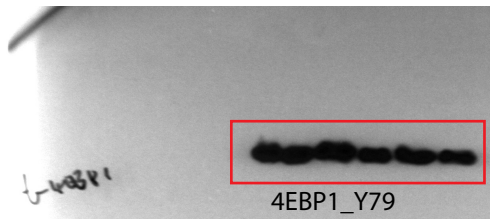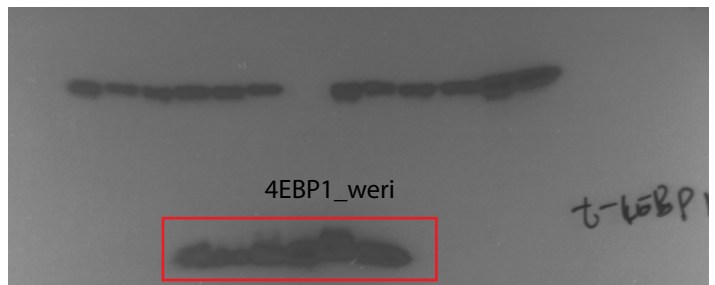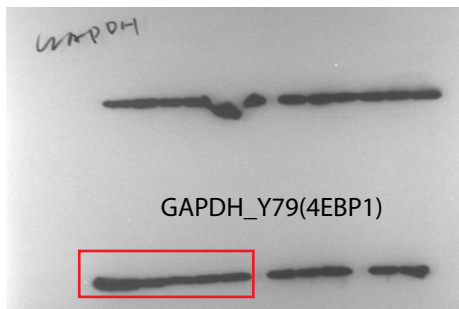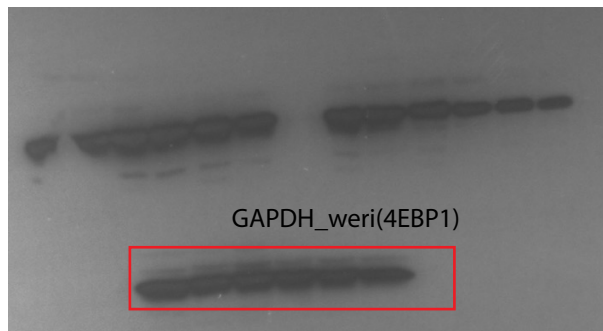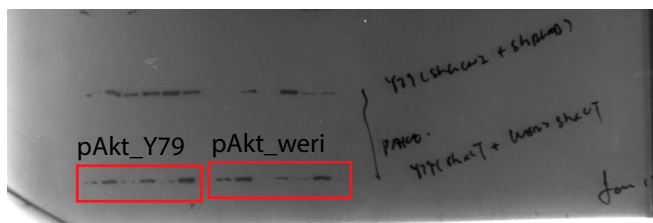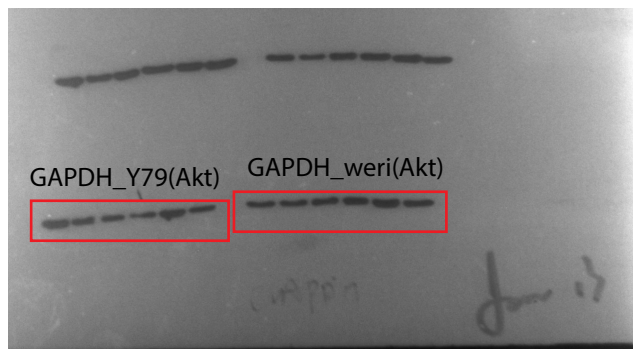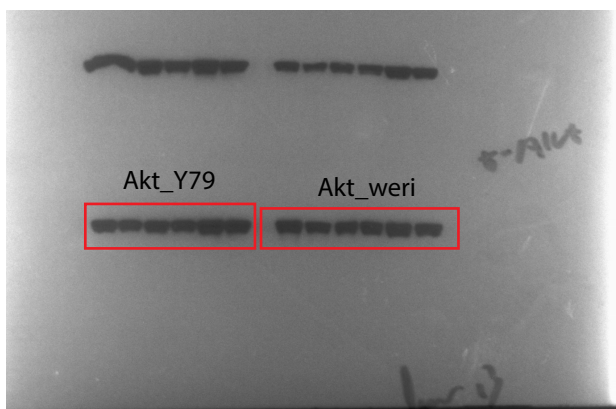

Supplement: Supplementary file 5 — Supplementary Material 5. [file 40170_2024_361_MOESM5_ESM.zip › Fig Suppl Fig4B_raw.pdf]
